# Modeling the chondrocyte-derived osteoblasts formation process reveals its molecular signature and regulation network

**DOI:** 10.1038/s41413-025-00500-6

**Published:** 2026-02-09

**Authors:** Raquel Ruiz-Hernández, Laurie Gay, Verónica Moncho-Amor, Pablo Martín, Jhonatan A. Vergara-Arce, Stefania Di Blasio, Thomas Snoeks, Unai Cossío, Ander Matheu, Maria M. Caffarel, Daniela Gerovska, Marcos J. Araúzo-Bravo, Amaia Vilas, Felipe Prosper, Sergio Moya, Daniel Alonso-Alconada, Ana Alonso-Varona, Gretel Nusspaumer, Javier Lopez-Rios, Karine Rizotti, Robin Lovell-Badge, Dominique Bonnet, Ilaria Malanchi, Ander Abarrategi

**Affiliations:** 1 Department of Cell Biology and Histology, Faculty of Medicine and Nursing, University of Basque Country (UPV/EHU), Leioa, Spain; 2 Center for Cooperative Research in Biomaterials (CIC biomaGUNE), Basque Research and Technology Alliance (BRTA), Donostia-San Sebastian, Spain; 3 Tumour-Host Interaction Laboratory, The Francis Crick Institute, London, UK; 4 Stem Cell Biology and Developmental Genetics Laboratory, The Francis Crick Institute, London, UK; 5 Biogipuzkoa Health Research Institute, San Sebastián, Spain; 6 Laboratory of Cancer Pathology, Center for Advanced Studies and Technology (CAST), University “G. D’Annunzio”, Chieti, Italy; 7 Biological Research Facility, The Francis Crick Institute, London, UK; 8 IKERBASQUE, Basque Foundation for Science, Bilbao, Spain; 9 Centro de Investigación Biomédica de fragilidad y envejecimiento, CIBERFES, Instituto de Salud Carlos III, Madrid, Spain; 10 Department of Hematology-Oncology, Instituto de Investigación Sanitaria de Navarra (IDISNA), CIMA Universidad de Navarra, Pamplona, Spain; 11 Centro de Investigación Biomédica en Red de Cáncer, CIBERONC, Instituto de Salud Carlos III, Madrid, Spain; 12 Centro Andaluz de Biología del Desarrollo (CABD), CSIC-Universidad Pablo de Olavide-Junta de Andalucía, Seville, Spain; 13 Haematopoietic Stem Cell Laboratory, The Francis Crick Institute, London, UK

**Keywords:** Bone, Bone quality and biomechanics

## Abstract

Endochondral ossification is a physiological process involving a sequential formation of cartilage and bone tissues. Classically, cartilage and bone formation have been considered independent processes at cellular level. However, the recently described multiple cell differentiation dynamics suggest that some bone cells are indeed the progeny of cartilage cells, or chondrocyte-derived osteoblasts. We hypothesized that the cartilage-to-bone phenotype transition is triggered by specific molecular events. First, the process was assessed in mouse bone tissue, and then, it was mimicked using in vivo cell implantation and in vitro serial differentiation protocols. Data indicates that cartilage cells transition to bone cell phenotype during postnatal physiological bone formation. This process can be reproduced using cartilage precursor cells coupled to specific implantation procedures or differentiation protocols. Gene expression profiling reveals that NOTCH, BMP and MAPK signaling pathways are relevant at the phenotype-switch, while the transcription factors *Mesp1, Alx1, Grhl3* and *Hmx3* are the feasible driver genes for chondrocyte-derived osteoblasts formation. Altogether, this report shows that endochondral ossification can be modeled using primary cell cultures and data indicate that this process is regulated by specific molecular events, previously described at skeleton morphogenesis during embryo development, and from now on also linkable to postnatal bone development and regeneration processes.

## Introduction

Stem cells are defined by their self-renewal and multilineage differentiation capacities. Postnatally, multiple types of lineage-restricted stem cells have been defined in different tissues and organs. The skeletal tissue stem cell lineage hierarchy includes a transition to progenitor stages able to differentiate into cartilage, bone, stroma, and adipose phenotypes.^1^ In mice, these stem and progenitor cells are located at the resting zone of the growth plate, periosteum, endosteum, bone marrow stroma, and perivascular niches, while several different markers, as Nestin, PTHRP, LEPR, CTSK, GLI1, FGFR3 or EBF3, have been used to identify each specific cell population in their compartment.^2–7^ Therefore, adult skeletal stem and progenitor cells are defined by their location, by their expression of different marker genes and by their multilineage differentiation capacity.

Endochondral ossification is the broadly accepted mechanism of long bone formation. Postnatally, stem cells located in the resting zone of the growth plate initiate cartilage differentiation. According to the classical definition, this is a terminal differentiation process in which calcified matrix-forming hypertrophic cartilage (HC) cells undergo apoptosis.^8^ Then, angiogenic type H vessels are formed, and the surface of the remaining calcified cartilage extracellular matrix is colonized by bone marrow-resident mesenchymal stem and osteoprogenitors to form bone tissue on it.^9^ According to this description, bone formation requires at least two independent skeletal stem cells, the first one at the growth plate resting zone to initiate chondrogenesis, and the second one at the bone marrow to form bone.

Following bone histology observations, Gerstenfeld and Shapiro questioned whether all growth chondrocytes have the same apoptotic developmental fate, and suggested that a subset of growth chondrocytes may progress to “some bone like phenotype”.^10^ Further on, seminal experimental reports based on reporter mice confirmed this hypothesis.^11–13^ Using Col2a1 reporter mouse embryos, the lineage tracing studies of growth plate hypertrophic chondrocytes indicated that at least some bone tissue cells were indeed formed from cartilage cells, and therefore, they did not undergo apoptosis. These works shifted the paradigm, because they provided the first effective evidence of the relevance of a cartilage-to-bone phenotype transition process in a typical physiological context.^14^ Thereafter, this phenomenon has been described using other reporter mouse models, always during embryonic development or perinatally,^15–22^ and even in bone fracture repair processes, in which endochondral ossification also occurs.^12,21,23–25^

According to these findings, bone formation during endochondral ossification may follow parallel processes, yielding chimeric bone formed by both bone marrow derived progenitors and phenotype-transitioned cartilage cells.^26^ Moreover, some reports suggest that hypertrophic chondrocytes give rise not only to osteoblasts, but also to other bone marrow-associated skeletal stem and progenitor cells and adipocytes.^27–29^ Published studies in this field are based on observations in reporter mouse models, and some are devoted to study the underlying molecular mechanisms.^30–32^ Interestingly, new in vitro cell culture and in vivo implantation methods are now available to induce bone formation, and they could be potentially used to address this process molecularly, including determining the identity of relevant transcription factors that direct the specific cell fates.

In this report, we aimed to investigate the specific molecular events regulating the formation of chondrocyte-derived osteoblasts. To this, we generated and used in vitro and in vivo tools capable of modeling the process, which enabled us to perform molecular studies and introduce specific manipulation to perform functional validations. First, using conventional histology techniques and samples at late developmental stages, we determine and assess that a subset of cartilage cells survives the HC and the extracellular matrix mineralization stage to further transition to the bone phenotype. Then, we model chondrocyte-derived osteoblasts formation by implementing in vivo cell implantation procedures that allow studies at intermediate differentiation stages. We also recapitulate chondrocyte-derived osteoblasts formation in vitro, which eases its characterization at molecular level, followed by in vivo cell implantation functional studies. In sum, here we introduce a series of modeling tools and methods that, together with reporter mouse models, helped us to define the molecular events triggering the chondrocyte-derived osteoblasts formation and to identify the key signaling pathways and transcription factors related to this process.

## Results

### Trabecular and cortical bone formation is a sequential process starting at the growth plate

**Fig. 1:**
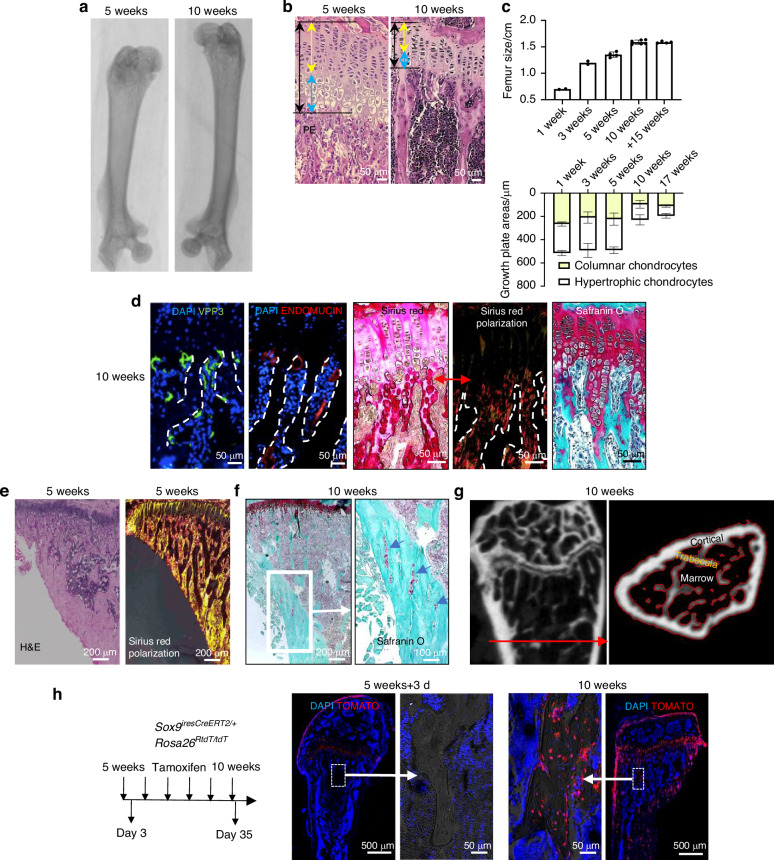
Postnatal endochondral bone development is related to growth plate cartilage cells. **a**–**g** Comparative images of 5- and 10-week-old mice, (5w, 5 weeks; 10w, 10 weeks) (*n* = 40). A) X-ray images of dissected femur bones. **b** Histology sections H&E Staining, (CC Columnar cartilage, yellow arrow, HC Hypertrophic cartilage, blue arrow). **c** Quantitative studies of bone size and size of the different growth plate areas during postnatal development (*n* = 2–5). **d** Histology characterization of growth plate area of 10-week-old mouse bone samples (Each specific staining is mentioned in each image. Safranin-O refers to Safranin-O/Fast Green staining. Dotted lines delimit trabecular bone and bone marrow). **e** Histology showing trabecular and cortical bone interconnected in 5-week-old mouse bone samples (Each specific staining is mentioned in each image), **f** Histology of 10-week-old mouse bone samples showing remains of cartilage matrix proteoglycan staining (Red at Safranin-O staining) at cortical bone. **g** micro-CT reconstruction of bone head area and a transversal section at red line, showing the interconnected nature of trabecular and cortical bone tissues. **h** Lineage tracing of SOX9^+^ cells. Schematic of tamoxifen treatment for the induction of tomato expression and representative images of histology immunostaining (*n* = 18)

Long bone samples from a common inbred mouse strain, C57BL/6, were used to study the morphological and cellular events at the hypertrophic chondrocyte-metaphyseal interface histologically (Fig. 1). During postnatal development, long bones continue to grow by endochondral ossification processes involving the growth plate and bone marrow areas (Fig. 1a). The architecture of these areas varies depending on the developmental stage. In mice up to 5 weeks of age, the resting zone, Columnar cartilage (CC) at proliferative zone and HC zone of the growth plate are easily identifiable by histology (Fig. 1b). However, at that stage, the primary spongiosa, which is the bone formation transition area located underneath the growth plate, is a network of closely interacting multilineage cells (Fig. 1b). Growth plate size is reduced from 5 to 10 weeks and later on its size is maintained, as observed at data from 17-week-old mice (Fig. 1b, c). The primary spongiosa morphology also changes and vasculature (Endomucin staining) and associated non-resorbing osteoclasts (VPP3 staining) near the growth plate limit are easier to identify (Fig. 1b, d).^9^ Additionally, incipient trabecular bone spicules surrounded by bone marrow areas are observed (Fig. 1d). Interestingly, at this stage it is evident that the cartilage is not resorbed evenly in all the front delimiting bone marrow and HC zones. Notably, the non-resorbed cells form bars of extracellular matrix, and Sirius red staining under polarized light shows some cells surrounded by fibrillar collagen (Fig. 1d, Fig. S1). Safranin-O cartilage proteoglycan specific staining shows the heterogeneity of the trabecular bone. Histology revealed presence of cartilage proteoglycans (red) at trabecular bone, corresponding to the remains of cartilage matrix with cartilage cells. The new bone matrix (blue) was presumably formed by bone marrow resident osteoprogenitors.

Interestingly, our histological staining suggests a continuum structure from the cartilage growth plate to the trabecular and cortical bone (Fig. 1e). Histologically, this observation is more evident in 5-week-old mouse samples, due to the abundance of the trabecular bone. In 10-week-old mice, the trabecular bone matrix is less prominent and therefore, conventional histology is not useful for observing the trabecular 3D structure or trabecular-cortical interconnection. However, cortical bone presented some elongated structures that were positively stained for cartilage proteoglycans, indicating the presence of non-resorbed cartilage extracellular matrix within cortical bone (Fig. 1f). Moreover, computed tomography studies revealed that the trabecular and cortical bones are indeed connected structures starting in the vicinity of the growth plate (Fig. 1g).

Sox9 is expressed at cartilage tissue cells (Fig. S2A). Lineage tracing data show that bone samples harvested 35 days after induction present tomato-positive cells in the trabecular and cortical bone of the metaphysis area, corresponding to chondrocyte-derived osteocytes at the newly formed bone tissue, generated by longitudinal bone growth during this period (Fig. 1h and Fig. S2C, D). Of note, Tomato positive cells are also observed at articular chondrocytes and more notably at perichondral cells which are known to contribute to bone formation during endochondral ossification.

### Chondrocyte-derived osteoblasts formation can be achieved in vivo using implantation procedures

**Fig. 2:**
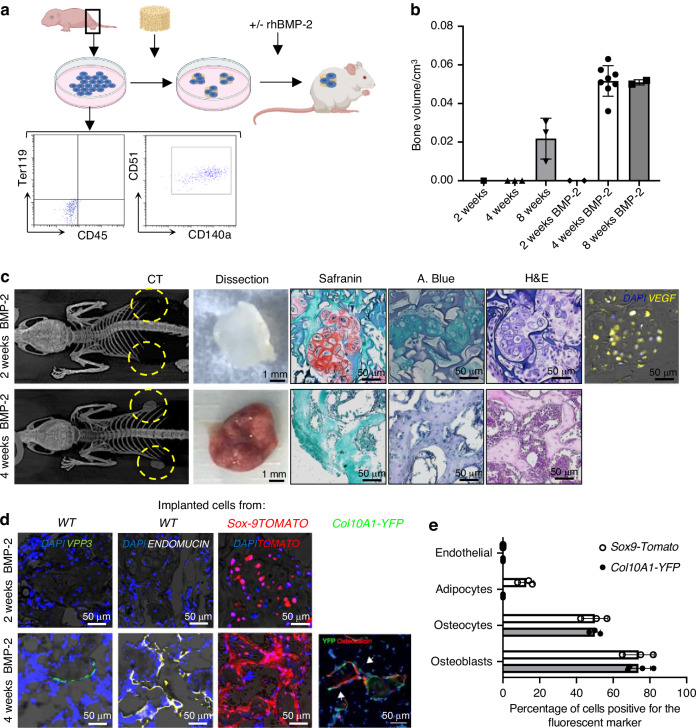
Ectopic implantation of Cartilage progenitors recapitulates endochondral ossification. **a** Schematic of cell harvesting, characterization by cell surface markers, seeding in scaffolds, rhBMP-2 addition, and subcutaneous implantation. **b**–**e** Sample characterization at 2-4-8 weeks after implantation (*n* = 2–8). **b** Quantification of ossicle volume by computed tomography (CT). **c** Computed tomography images (CT, yellow circles indicate implant location) followed by gross morphology of dissected samples and details of conventional and tissue-specific histology staining (Red color at Safranin staining, and Blue color at Alcian Blue (A. Blue) staining, indicate cartilage extracellular matrix proteins; H&E, Hematoxylin and Eosin) and VEGF immunostaining. **d** Immunostaining to locate osteoclasts (VPP3), Vasculature (ENDOMUCIN) and implanted cells (TOMATO or YFP as indicated by white arrows). **e** Quantification of implanted cells within the newly formed cell phenotypes in 4-week samples (From tomato or YFP immunostaining. “Sox9-Tomato”, quantification from Tomato immunostaining at scaffolds implanted with tamoxifen-treated cells obtained from tomato reporter mice; “Col2A1- YFP”, quantification from YFP immunostaining at scaffolds implanted with cells extracted from Col2A1- YFP reporter mice)

Cartilage progenitor cells from mice of different genotypes were extracted from long bone heads of mouse pups, characterized by cytometry, seeded in a gelatin scaffold with rhBMP-2 and further implanted subcutaneously in immune-compromised hosts (Fig. 2 and Fig. S3). Cells were those retrieved from control C57BL6/J mice; tomato reporter mice and Col2A1-YFP reporter mice, both treated with Tamoxifen to express lineage traceable fluorescent proteins under the control of Sox9 promoter.

Data indicates calcified tissue formation at implants, which was detectable and measurable by computerized tomography (Fig. 2). Implants with rhBMP-2 formed subcutaneous ossicles after 4 weeks, while the same implantation procedure without rhBMP-2 yielded calcified tissue only after 8 weeks of implantation (Fig. 2b and Fig. S4). 2 weeks after implantation with rhBMP-2, hypertrophic VEGF-producing cartilage cells were observed, with no vascularization or osteoclast activity (Fig. 2c). Thereafter, at 4 weeks, the cartilage was replaced by bone tissue, with vascularized mature hematopoietic bone marrow and osteoclasts at the bone surface (Fig. 2c–e, Fig. S4). The scaffolds implanted with cells expressing tomato reporter show tomato positive cartilage and bone cells, and also adipose and bone lineage cells (Fig. 2d, e). However, the scaffolds implanted with Col2A1-YFP reporter cells show a more specific contribution to bone tissue formation, and did not give rise to adipocytes (Fig. 2d, e). Note in all cases the newly formed tissue is a chimeric tissue with contribution of host cells also participating in the tissue formation process (Fig. 2e).

Taken together, these findings indicate that the implanted cartilage-progenitor primary cells participate in the tissue formation process, and that cartilage-to-bone phenotype transition occurs between 2 and 4 weeks after implantation.

### Chondrocyte-derived osteoblasts formation can be modeled in vitro

**Fig. 3:**
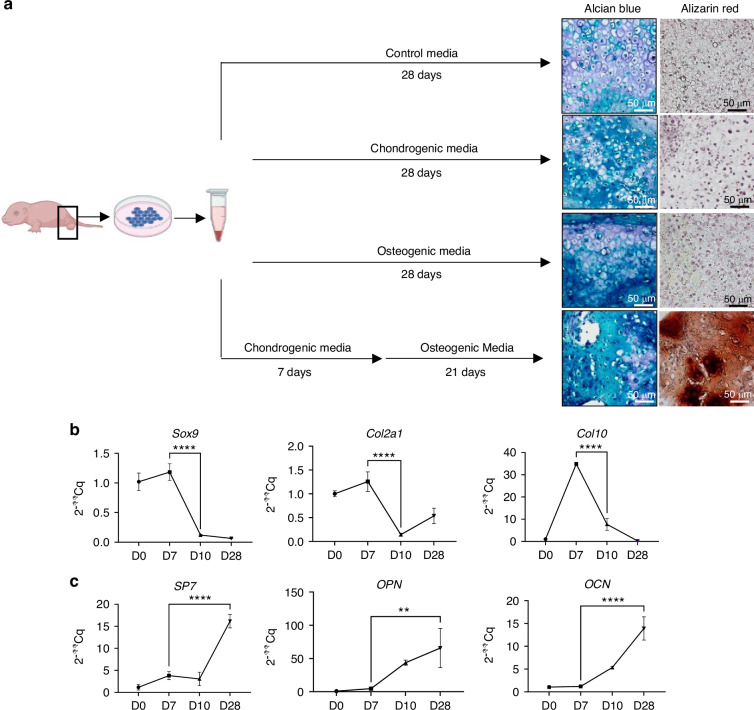
Primary cartilage precursor pellet cell culture and serial differentiation yields calcification. **a** Schematic of cell-harvesting and pellet formation, followed by multiple control, chondrogenic or osteogenic treatment sequences. Histology images of tissue specific staining (Alcian Blue, Alizarin red) (*n* = 10). Gene expression kinetics of specific **b** cartilage, or **c** bone, tissue markers on samples treated with chondrogenic media for 7 days, followed by osteogenic media treatment (*n* = 9)

Aiming to mimic chondrocyte-derived osteoblast formation process, the same source of primary cells was used in in vitro cell culture experiments. To do it, cells were induced to chondrogenic differentiation, followed by osteogenic differentiation. Assays were done in cell pellet conditions, which is the most common in vitro cartilage differentiation approach (Fig. 3), and also in scaffolds similar to the implantation approach (Fig. S5A). Multiple additional differentiation conditions were tested as controls, and samples were assessed by histology for extracellular matrix cartilage glycosaminoglycan (GAG) staining (Alcian blue staining), extracellular matrix calcification (Alizarin red staining), and gene expression studies (Fig. 3).

Cell culture conditions were optimized in preliminary assays by testing different timings for each differentiation media, until we get the selected protocol of 7 days at chondrogenic media and 21 days at osteogenic media as the optimal one to obtain calcification. Histological data related to cell morphology and alcian blue staining show cartilage-like cells in all media treatment, indicating these primary cells have a basal cartilage commitment (Fig. 3a). Cell pellets treated with osteogenic media did not differentiate into calcified bone and extracellular matrix, surely because these cells were chondrogenic progenitors programmed only for chondrogenic differentiation (Fig. 3a). Interestingly, the cell pellets induced to cartilage differentiation first and further treated with osteogenic media showed smaller cell size and were the only ones with calcified extracellular matrix (Fig. 3a). For this treatment condition we explored the gene expression kinetics of selected cartilage and bone tissue marker genes, and data indicate an initial increase of cartilage genes, followed by further reduction of them and increase in expression of bone marker genes (Fig. 3b). For comparative purposes, gene expression data were also obtained from cell-pellets treated with the other control treatments (Fig. S5B), which confirmed that the gene expression pattern of serial differentiation protocol was unique. Altogether, data suggest that chondrocyte progenitors require some maturation towards cartilage differentiation to gain the competence of further matrix calcification ability.

### The chondrocyte-derived osteoblasts formation is regulated by specific molecular mechanisms

RNAseq studies were carried out on pellet samples harvested at different cell culture time points, specifically: 1. at the end of the cartilage priming process (7 days); 2. 1 and 3 days after the switch to osteogenic induction (day 8 and 10), and 3. at the endpoint of osteogenic differentiation (Day 28).

**Fig. 4:**
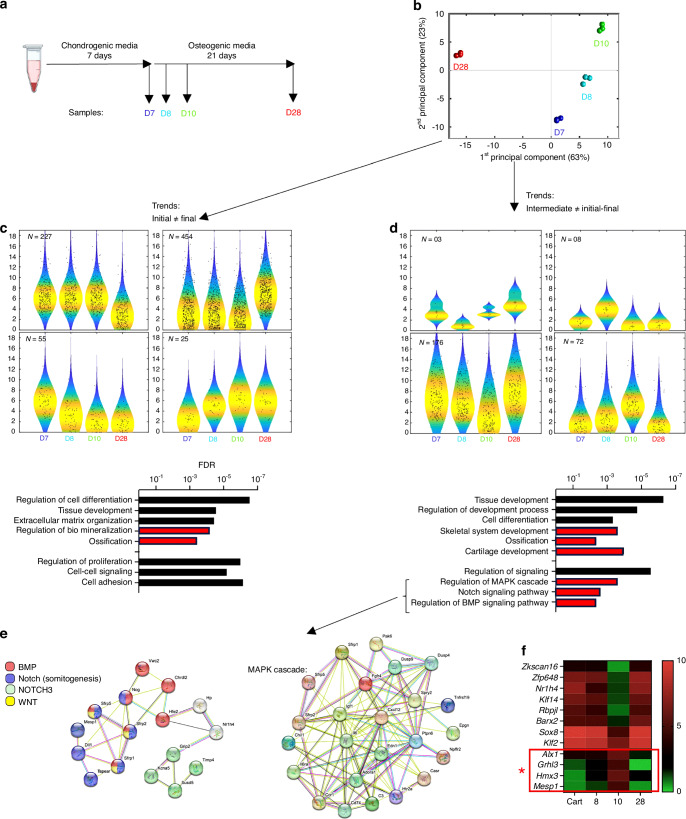
In vitro detection of the molecular signature of Cartilage-to-bone phenotype switch by RNAseq study. **a** Schematic of the assay. **b** Principal component analysis of samples harvested at different points of the serial chondrogenic-osteogenic differentiation protocol (D7, day 7 of culture and before changing to osteogenic media; D8, Day 8; D10, Day 10; D28, Day 28) (*n* = 3). **c** Trend clusters and gene ontology study corresponding to the analysis of the pool of genes differentially expressed at the end of the study compared to the end of chondrogenic differentiation (D28 vs D7). *N* in the graphic correspond to genes observed in each cluster. **d**–**f** Data from trends at intermediate points. **d** Trend clusters and gene ontology study corresponding to the analysis of the pool of genes differentially expressed at the beginning of osteogenic induction. *N* corresponds to genes observed in each cluster. **e** Transcriptional regulatory networks of genes related to signaling pathways. **f** Heat map of transcription factors modulated at intermediate points (*, Highly expressed only at intermediate points)

Principal component analysis revealed clear differences in gene expression at the selected time points, suggesting global changes in gene expression just after the change on culture media and therefore at the beginning of the cartilage-to-bone phenotype switch (Fig. 4b). Gene expression trend cluster maps were generated and classified (Fig. 4c, d). In the first group, those trends defining the gene expression change between initial cartilage (D7) and final bone phenotype (D28) were grouped and subsequently analyzed (Global trend study at Fig. 4c). A gene ontology study of these genes revealed that cells undergo ossification by mineralization of extracellular matrix (Fig. 4c). A second group was formed by those trends defining the gene expression changes during the initial transition stages (genes with altered expression at D8 and D10) (Fig. 4d). This set of genes also indicated a regulation in skeletal cell differentiation. Interestingly, the MAPK cascade, NOTCH signaling pathway and BMP signaling pathway, were found to be the molecular events most modulated during the initial stages of cartilage-to-bone transition (Fig. 4d). A transcriptional regulatory network analysis (Fig. 4e) indicated that some of these genes are related to somitogenesis, an embryonic developmental process that gives rise to skeletal tissues, muscle, endothelial cells and dermis, suggesting that there is a molecular resemblance between cartilage-to-bone phenotype transition and other developmental processes. The transcription factors altered at the initial stages of cartilage-to-bone transition were identified. (Fig. 4f).

Altogether, using in vitro cell culture approaches, we have obtained gene expression data characterizing the chondrocyte-derived osteoblasts formation process. We have also identified the presence of transcription factors relevant for skeletal morphogenesis during embryonic development.

### Cartilage-to-bone phenotype switch related transcription factors are expressed at bone forming locations

**Fig. 5:**
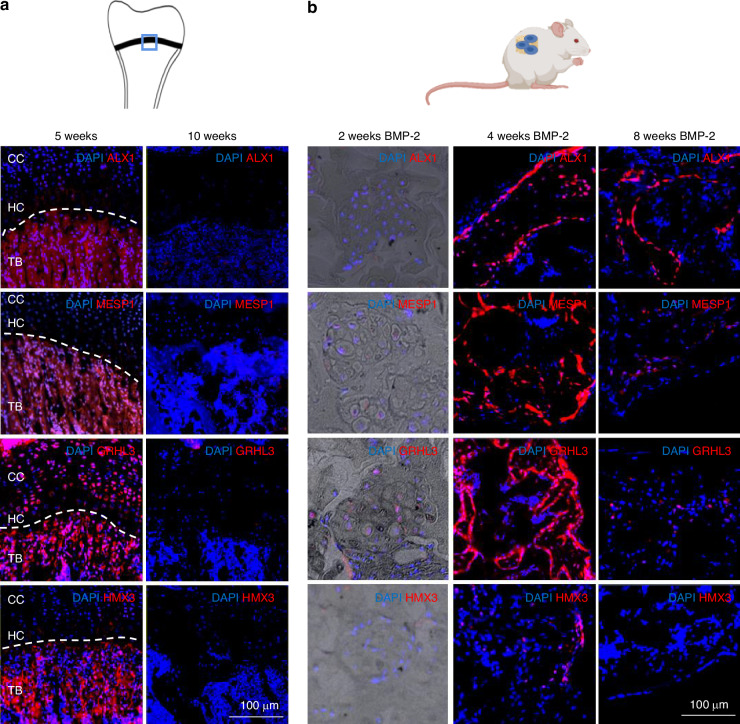
Targeted transcription factors are expressed at bone formation areas and times. **a** Immunostaining at bones during postnatal development. Blue square at bone schematic indicates the area shown in the images. Samples from 5- and 10- week-old mice were analyzed (CC columnar cartilage, HC Hypertrophic cartilage, TB Trabecular bone area) (*n* = 3). **b** Immunostaining at bone-forming implantation model. Samples harvested at 2, 4, and 8 weeks of implantation (*n* = 3)

To validate the data obtained in the in vitro gene expression study, the presence of the observed transcription factors in vivo was evaluated by immunostaining (Fig. 5). First, histological samples from late postnatal bone development were tested (Fig. 5a). All four transcription factors were found in the primary spongiosa area of samples from 5-week-old mice, the area in which cartilage-to-bone phenotype switch starts. Interestingly, Mesp1, Alx1 and Hmx3 were not present in the growth plate, indicating that they were not relevant in the chondrogenesis process. In samples from 10-week-old mice, there was no expression of these transcription factors in the growth plate or bone marrow areas (Fig. 5a).

Samples from the subcutaneous implantation-based in vivo endochondral ossification model were also evaluated for the presence of these transcription factors at different timepoints after implantation (Fig. 5b and Fig. S6). Interestingly, the samples harvested 2 weeks after implantation showed cartilage formation and no staining for the four transcription factors. In contrast, in samples harvested at 4 weeks after implantation, previously shown to be the time point for cartilage-to-bone phenotype switch in this model (Fig. 2c), we could observe here that implanted tomato positive cells show also nuclear positive staining for Mesp1, Alx1 and Grhl3 transcription factors at this timepoint (Fig. S6). Late in the bone formation process (at 8 weeks), the expression of the targeted genes disappears again, as observed in samples harvested 8 weeks after implantation, when the de novo formed bone reaches homeostasis similarly to an adult bone (Fig. 5b). Alx1 is still present, which may be still expressed at a lower amount at 8 weeks or just degraded at lower rate by cells.

The next step was gene silencing and overexpression in chondrocyte progenitors. In vitro testing indicated that specific gene silencing of any single factor leads to abrogation of in vitro bone formation (Fig. S7). When *Alx1* and *Mesp1* were silenced, there was non-significative effect in the expression of other genes. When *Hmx3* and *Grhl3* were silenced, the rest of the target genes were also downregulated (Fig. S8). Conversely, when gene overexpression was tested, the in vitro differentiation ability was not altered in any case. Moreover, when any of them is upregulated, at in vitro differentiation procedure the rest of the selected genes tend to be upregulated too, which may indicate a boost and speed up at the tissue formation process in all cases (Fig. S9).

**Fig. 6:**
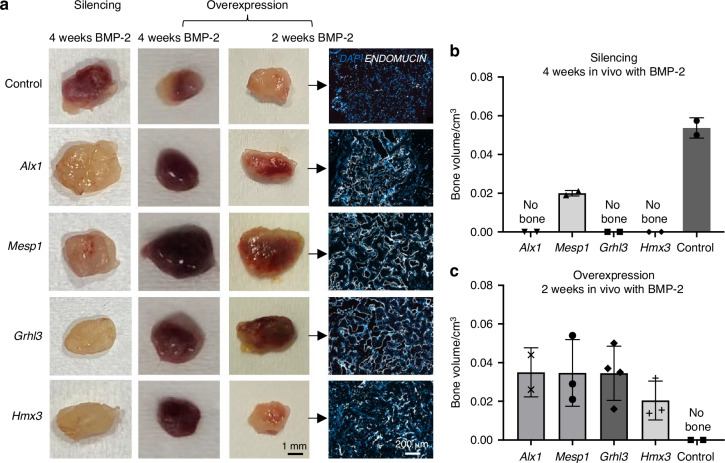
Gene silencing or overexpression of selected genes modify bone formation. **a** Macroscopic appearance of scaffolds at harvesting. Immunostaining of Endomucin and DAPI at samples implanted 2 weeks with BMP-2 (*n* = 2–5). **b**, **c** Ossicle volume measured by Computed tomography. **b** Ossicle volume in samples carrying gene silenced cells and BMP-2, and implanted for 4 weeks. **c** Ossicle volume in samples carrying specific gene overexpressing cells and BMP-2, and implanted for 2 weeks

Functional analysis of candidate transcription factors was performed using the subcutaneous implantation-based ossification model to test whether these transcription factors are required for in vivo bone formation. Gene silencing abrogated bone formation at 4 weeks in all cases, as observed by gross morphology (Fig. 6a) and by quantification of computerized tomography data (Fig. 6b), suggesting all 4 genes are relevant and each one of them necessary in tissue formation process. In contrast, cells overexpressing each one of the selected genes produced calcified ossicles of equivalent size to those of the control samples at 4 weeks, but more vascularized. Assays reducing the implantation time to 2 weeks were also performed. At this timepoint, and in contrast to control implants, the scaffolds with cells overexpressing the target genes exhibited vascularized and calcified ossicle formation, as observed by gross morphology (Fig. 6a) and by quantification of computerized tomography data (Fig. 6c). Histology of these samples indicated no vascularization or bone formation in control samples at this 2 weeks timepoint, but highly vascularized bone marrow formation in scaffolds implanted with cells overexpressing *Alx1*, *Mesp1* and *Grhl3* (Fig. 6a).

These data indicate these 4 transcription factors discovered as relevant in bone formation process are indeed correlated, but each of them has irreplaceable activity during bone formation process. Silencing of each one of them abrogates tissue formation process, while overexpression of any of them triggers vascularization and bone formation processes.

## Discussion

Chondrocyte-derived osteoblasts have been discovered and studied mainly using in vivo transgenic mouse models. In this work, we explore the possibility to generate cell culture tools and apply them to this field, aiming to facilitate its study by in vitro gene expression and in vivo implantation approaches. Inspired in tissue engineering and biomaterials-based approaches, this work takes advantage of a relatively simplistic in vitro 3D culture model useful to generate relevant molecular data, and to validate the findings by in vivo functional assays.

First, bone tissue samples were studied. Classically, trabecular and cortical bone are defined as separate bone entities with distinct extracellular matrix and structure. Cortical bone is defined as compact-dense bone found at the outer layer of the long bones, while trabecular or cancellous bone is defined as a porous network located mainly in the metaphysis bone marrow and with high remodeling activity. The metaphysis area of developing bones is a dynamic structure and bone samples in young adults provides valuable information about trabecular bone formation. In these samples, blood vessels and vessel-associated osteoclasts invade the empty spaces left by apoptotic hypertrophic cells. Using histology and 3D imaging, and in contrast to the well-established apoptosis-based endochondral ossification model, here we show that some cartilage cells remain embedded in the trabecular bone matrix, and they are able to modify the extracellular matrix, in example, collagens (Fig. 1 and Fig. S1).

However, whether these cartilage cells die or survive bone remodeling cannot be elucidated using conventional 2D histology or 3D imaging techniques in non-transgenic mice. Instead, transgenic mice bearing the combination of site-specific recombination systems (Cre-loxP) with conditional reporter genes are useful to label and lineage tracing tissue-specific cells without disturbing their physiological functions.^33^ Previous reports already used this tool to track mesenchymal cell fate in bone and cartilage development context.^5–7,12,17–20,25,34–38^ Here we focused on Sox9, the master regulator of chondrogenesis, which at mice bone organ is found only in cartilage cells located at the growth plate of long bones (Fig. S2). Therefore, and similar to Banali et al.,^39^ we focused on tracing in juvenile mice the fate of Sox9 positive growth plate cartilage cells (Fig. 1 and Fig. S2). These data assess the relevance of the chondrocyte-derived osteoblasts in physiological context, as we found that the derivatives of a subset of growth plate cartilage cells survive within the trabecular bone matrix, and transition their phenotype to bone cells.^11,17^

Working with endogenous bone tissue helped us to reveal chondrocyte-derived osteoblasts at postnatal stages and in young adults. Most likely, chondrocyte-derived osteoblasts coexist with the classically defined cartilage apoptosis and replacement by bone marrow resident osteoprogenitors, resulting in the formation of trabecular and cortical bone tissues of chimeric cellular origin.^26^

At this point, we wondered whether it would be possible to define the underlying molecular mechanisms of the chondrocyte-derived osteoblasts formation process. To do so, we looked for modeling options compiling cultured cells and implantation procedures, which would facilitate the understanding of the molecular mechanisms involved.^40^ Primary cartilage-progenitors were selected as a feasible cell culture model, so, cartilage progenitor cells were extracted from long bone heads of mouse pups.^41,42^ This approach yielded a homogeneous Ter119^-^, CD45^-^, PDGFR^+^, and CD51^+^ cell population (Fig. 2 and Fig. S3), in accordance with the skeletal progenitor cell phenotype previously reported by Chan et al.^7^ These cells were used in a bone forming implantation model based on endochondral ossification stimulation by the rhBMP-2 osteogenic protein.^43–50^ This model induces bone formation following consecutive cartilage and bone differentiation processes, and data shows that the implanted cells participate in these tissue formation processes. Moreover, cartilage-to-bone phenotype transition occurs between 2 and 4 weeks after implantation (Fig. 2 and Fig. S4).

To model and study cartilage-to-bone transition, we mimicked the process in cell culture context (Fig. 3 and Fig. S5). The multilineage potential of mesenchymal cells is usually assessed in vitro by using specific differentiation media and specific cell culture conditions, involving cell pellet formation for optimal cartilage differentiation. To recapitulate the chondrocyte-derived osteoblasts formation process, a serial differentiation protocol was used in which primary progenitor cell pellets were first lineage-committed to cartilage, and subsequently treated with osteogenic media. Interestingly, chondrogenic priming is a concept previously explored to effectively boost in vivo bone formation by implantation of previously in vitro primed, mesenchymal stem cells toward cartilage differentiation.^51,52^ However, to our knowledge, a serial in vitro differentiation approach has not been used to study cartilage-to-bone transition point. Data shown in Fig. 3 strongly suggest that chondrocyte progenitors being committed to cartilage differentiation are those which gain the competence to acquire later on the osteogenic features.

Being able to model the chondrocyte-derived osteoblasts formation at cell culture context allowed us to study this process at a molecular level. Gene expression studies shown at Fig. 4 indicate that the in vitro serial differentiation protocol induced the expression of genes related to skeletal cell differentiation and ossification by mineralization of extracellular matrix. Moreover, there were regulated multiple genes related to the MAPK cascade, NOTCH signaling pathway and BMP signaling pathway, which are well-known regulators of skeletal development and homeostasis.^53,54^

Focused on transcription factors, it was observed that there were no cell reprogramming-related transcription factors, suggesting that the cartilage-to-bone transition is a phenotype switch process, rather than a dedifferentiation or reprogramming event. Indeed, most of transcription factors found in the study have been previously related to bone and cartilage tissue morphogenesis. Among downregulated ones, zfp648 and Barx2 are related to collagen 2 and cartilage tissue.^55,56^ Moreover, Barx2 has been previously reported as co-expressed with Sox-9, and they cooperate in regulating the expression of collagen 2. Klf14 inhibits osteogenesis and deficiency in Nr1h4 is related to increased osteoclastogenesis.^57,58^ Therefore, the reduction in the expression of these transcription factors suggests a reduction in chondrogenesis process. On the other hand, among the 4 transcription factors highly expressed (Fig. 4f), *Alx1*, also known as cartilage homeoprotein 1 (*Cart1*) is an evolutionarily conserved regulator of embryonic skeletal morphogenesis, especially craniofacial bone development,^59^ which is highly expressed in early chondrocytes of primary primordia, but expressed at lower levels by mature chondrocytes,^60^ and upregulated during the MSC osteodifferentiation process.^61^ *Hmx3* is required for the morphogenesis of the developing inner ear. Depending on the gene mutation approach, *Hmx3* null mice are viable,^62^ or die postnatally, with growth retardation during their postnatal development, which is also reported as postnatal Dwarfism.^63^ *Grhl3* has been defined as an important gene in palatogenesis and cranial development and it has been related to craniosynostosis.^64^ Finally, *Mesp1* is considered a master regulator of the mesoderm differentiation. In skeletal tissue, *Mesp1* has been related to skull bone formation.^65^ Different lineage tracing approaches have identified chondrocytes, adipocytes, bone precursors and bone marrow mesenchymal stroma cells, among others, as the progeny of MESP1 positive cells.^66,67^ Altogether, their roles in chondrogenesis and osteogenesis, along with the specific expression patterns observed, were compatible with the starting of the switch from cartilage to bone phenotype when chondrocyte progenitors are primed to undergo ossification at the formation point of trabeculae underneath the growth plate. Note too that bone vascularization is a key process during endochondral ossification, and it is taking place at the same location, while interestingly, *Grhl3*, *Mesp1* and *Alx1* have been related to vascularization in different contexts.^9,68–70^ To validate the in vitro results, the overexpressed transcription factors were studied in vivo. We found that they are expressed during the endochondral ossification process, specifically after cartilage formation and during the transition to the bone phenotype (Fig. 5 and Fig. S6). Further functional analysis also shows that silencing of these transcription factors functionally impairs the bone formation process, while their overexpression speeds up this and vascularization (Fig. 6 and Figs. S7, 8). To our knowledge, there is no known direct, well-established interaction between these specific genes, but they do play relevant roles in overlapping developmental pathways and developmental stages. Our data suggests that, their coordinated expression and function may be indispensable for successful cartilage and bone tissue formation and vascularization.

We acknowledge that this research may show several limitations. First, focused on translational value of results and conclusions, we should note that the growth plate closes after puberty in humans, and therefore, any result obtained in mouse models may be limited to certain species. Also, primary cultures may quickly lose their cellular identities. Precisely, here presented protocols try to avoid it by inducing immediately the chondrogenic cell commitment, thereby standardizing the phenotype at the cell culture and preparing them to be able to mimic the cartilage-to-bone phenotype switch. Also note that, at in vitro cell cultures, multiple differentiation media are used as cell-extrinsic features or signaling, which indeed may not reflect accurately the cellular and signaling complexity of in vivo environment. Of note, other factors such as blood vessel endothelial cells and hematopoietic cells as osteoclasts may certainly interact with, or even drive, the normal endochondral ossification processes and that effect is not tested in these in vitro studies. Indeed, the transcription factors observed are related to ossification, but also to induce osteoclasts, induce vascularization and reduce chondrogenesis. We consider that data assessment by in vivo implantation models has been pivotal to define the relevance of the findings. Gene silencing strategy abrogates in vivo bone formation, but it may be possible that it even abrogates chondrogenesis, vascularization or clastogenesis, none of them can be discarded with these results. Besides, gene overexpression hints at a positive correlation between vascularization and bone formation, but the mechanism in which the implanted cells induce the vascularization directly or indirectly, and which other cells are involved, has not been addressed in this study. Moreover, the cell implantation model here presented is very different from the actual development of a long bone, and conclusions raised from here may need to be further assessed using genetic KO mouse models.

In sum, histology of postnatal long bone tissue development indicates that the remodeling of HC does not occur evenly at the cartilage vs bone-marrow interface, leading to the formation of cartilage columns which eventually become trabecular bone. Cellular lineage tracing studies have shown that growth plate cartilage, trabecular bone and cortical bone are indeed continuous structures at a cellular level, in which cartilage-to-bone cell phenotype switch occurs, with the subsequent gradual modification of extracellular matrix components. Using a Sox9 lineage tracing model we confirmed that this existence of transition occurs during postnatal bone growth. We also showed that it can be replicated in an in vivo extramedullary bone formation model, using chondrocyte progenitors implanted cell-carrier 3D scaffolds. Strikingly, this work shows that chondrocyte-derived osteoblasts formation can be mimicked using in vitro cell culture approaches, which can replicate the sequential chondrocyte to bone differentiation of endogenous bones. This allowed us to define molecular events involved in this process and to identify the transcription factors Alx1, Mesp1, Grhl3, and Hmx3 as its key regulators. These transcription factors are expressed in vitro at cartilage-to-bone phenotype switch point, and in vivo in the areas associated with bone formation, such as developing postnatal bone, which is recapitulated in ectopic bone-forming implants. Using the latter, we functionally showed that Alx1, Mesp1, Grhl3, and Hmx3 transcription factors are indeed essential during postnatal bone development.

## Materials and methods

### Extended materials and methods

See at the supplementary file the extended detailed description of materials and methods, including the commercial references of all reagents at Table S1.

### Animals

All animal experiments were approved by the ethical committee and local competent authorities, under the project licenses no. 70/8560, PP8826065 and PRO-AE-SS-171 (competent authorities: Departamento de promoción económica, medio rural y equilibrio territorial, Diputación de Gipuzkoa, and UK Home Office animals (scientific procedures) act 1986).

### Bone tissue characterization

Tibia, femur and implant samples were imaged by computed tomography (CT, Skyscan 1176, Bruker) or fluorescent stereoscope, followed by fixation and routine histology, compiling decalcification with Osteosoft Mild decalcifier solution, paraffin embedding and sectioning at 4 µm using a microtome (HistoCore BIOCUT Manual Rotatory Microtome, Leica Biosystems). Sections were used for staining and immunostaining. Images were obtained in optical and confocal microscopes.

### Primary cartilage progenitor cell harvesting

Murine cartilage progenitors were obtained from different mouse strains. Cells were isolated from 5 or 6 days-old pups following the procedure described by Gosset et al. and Salvat et al.^41,42^ A fraction of these cells was separated and characterized by Flow Cytometer. The rest was frozen down for further studies.

### Scaffold preparation

Cell carrier implants were prepared as previously described.^46,48^ Briefly, 20 × 60 × 7 mm Gelfoam gelatin sponges (Pfizer, Cat. #0009-0323-01) were sectioned into 128 pieces (4 × 4 × 4 mm each), washed with ethanol 70% (Scharlab, Cat. #ET0003005P) and rehydrated in sterile phosphate buffered saline (PBS) (Gibco, Cat. #14040). Cells were diluted in culture media at 1 × 10^6^ cells/mL and 100 μL (1 × 10^5^ cells) were carefully inoculated in each scaffold using a 1 mL syringe (Braun, Cat. #9161406 V) with 25 G needle (Braun, Cat. #9186166). Cell-seeded scaffolds were transferred to polystyrene ultra-low attachment 24-well plates (Corning, Cat. #3473) and kept in cell culture conditions for 3–5 h. Then, culture media was added, and scaffolds were maintained in cell culture conditions for further studies.

### In vivo subcutaneous implantation

Cell-seeded scaffolds cultured during 3–7 days were transferred to clean well and wet with 5 μL of 5 μg/μL rhBMP-2 (Noricum, Cat. #rhBMP-2) reconstituted in 50 mmol/L acetic acid (Fluka, Cat. #27225). Then, 30 μL of 2% CaCl_2_ reconstituted thrombin from human plasma and 30 μL of water reconstituted fibrinogen from human plasma were incorporated. Clotting was allowed during 10 min in cell culture conditions before proceeding with in vivo implantation subcutaneous at ten-week-old healthy NOD-SCID mice as previously described.^45,48^ At endpoint, Carbon dioxide (CO_2_) overdose was used to euthanize the animals.

### In vitro cell culture studies

Self-assembled cell pellets were formed by centrifugation of 2.5 × 10^5^ to 1 × 10^6^ cells at 200 *g* for 5 min. Long-term cell cultures were done in cell pellets and cell-seeded scaffolds. Differentiation media were: MesenCult-ACF Chondro Differentiation Kit; MesenCult Osteo Stimulation Kit. At stablished endpoints, samples intended for histological analysis were fixed and processed for paraffin-embedding and staining as described above, while samples intended for gene expression studies were processed as follows.

### Gene expression studies

Cell pellet samples were disrupted and homogenized before RNA isolation. Quantitative Real-Time PCR (qPCR) and RNA-sequencing assays were performed, followed by RNA-sequencing data analysis. See details at the Supplementary File.

### Cell transfections

Cell transfection was performed with lentiviral particles. Gene silencing tools were obtained at Santa Cruz, while the design and production of lentiviral vectors for the stable transgene expression were achieved by GEG Tech. See details at the Supplementary File.

### Statistics

Data are presented as mean ± SD, and each individual point is provided in the plots. *P* values of less than 0.05 were considered statistically significant (ns *P* > 0.05; *P < 0.05; **P < 0.01; ***P < 0.001).

## Supplementary information

Supplemental material

## Data Availability

The GEO series record GSE243090 provides access to RNAseq data. https://www.ncbi.nlm.nih.gov/geo/query/acc.cgi?acc=GSE243090.
