# Supplementary material for: Modeling the chondrocyte-derived osteoblasts formation process reveals its molecular signature and regulation network
Source: Bone Res. 2026 Feb 9;14:19. doi: 10.1038/s41413-025-00500-6 (PMC12886771; doi:10.1038/s41413-025-00500-6)
Supplement: Supplementary file 1 — Supplemental material [file 41413_2025_500_MOESM1_ESM.pdf]

Supplemental information for:

Modeling the Cartilage-to-Bone transition process reveals its molecular signature and regulation network.

Raquel Ruiz-Hernández[1,2], Laurie Gay[3], Verónica Moncho-Amor[4,5], Pablo Martín[1,2], Jhonatan A. Vergara-Arce[2], Stefania Di Blasio[3,6], Thomas Snoeks[7], Unai Cossío[2], Ander Matheu[5,8,9], Maria M. Caffarel[5,8], Daniela Gerovska[5], Marcos J. Araúzo-Bravo[1,5,8], Amaia Vilas[10], Felipe Prosper[10,11], Sergio Moya[2], Daniel Alonso-Alconada[1], Ana Alonso-Varona[1], Gretel Nusspaumer[12], Javier Lopez-Rios[12], Karine Rizotti[4], Robin Lovell-Badge[4], Dominique Bonnet[13], Ilaria Malanchi[3], Ander Abarrategi[1,2]*

Supplementary figures 1 to 9

Extended Materials and Methods:

Supplementary Tables 1 and 2

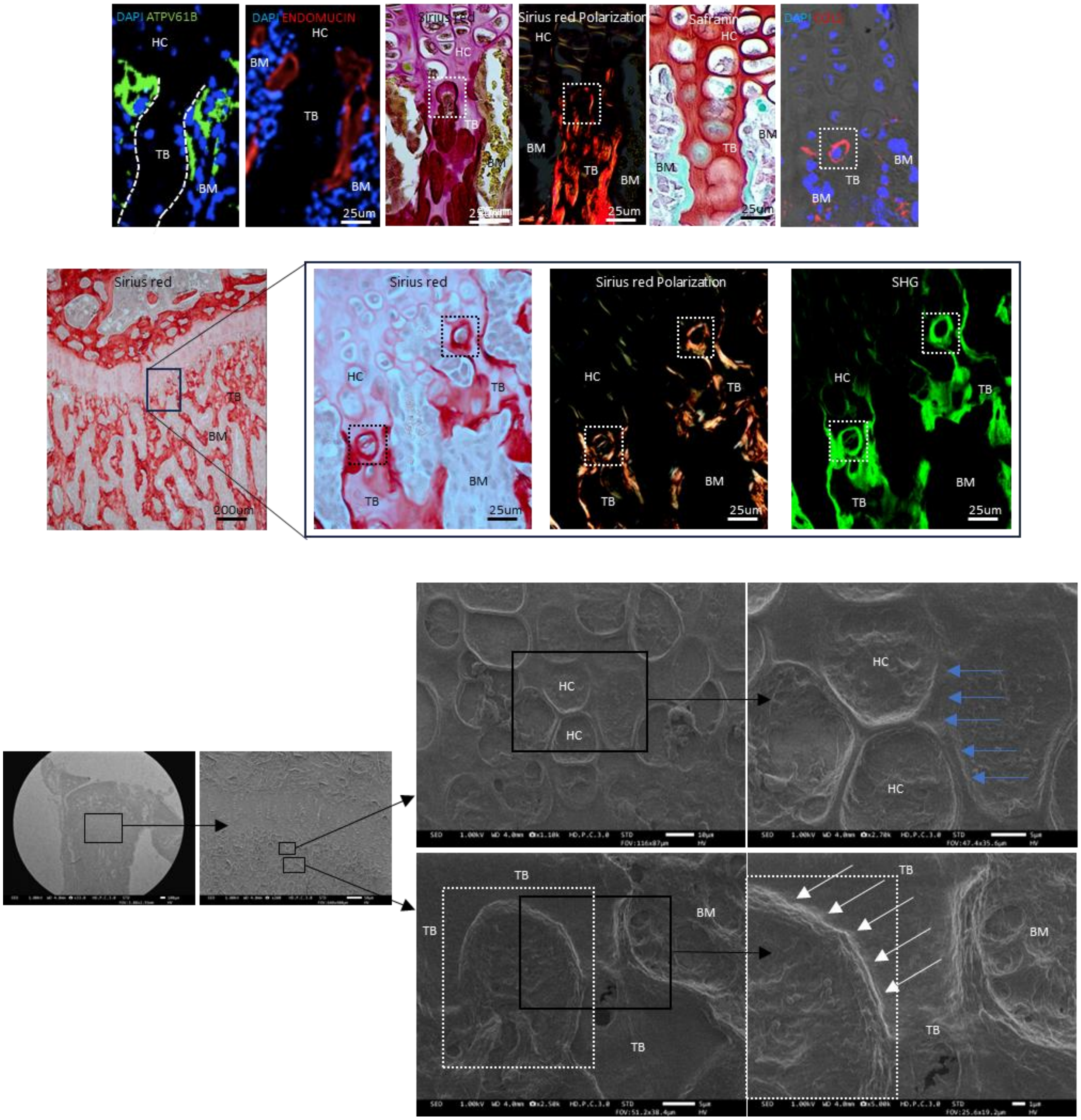
DAPI ATPV61B
HC
TB
BM
DAPI ENDOMUCIN
HC
BM
TB
BM
25um
Sirius red
HC
TB
BM
Sirius red Polarization
HC
BM
TB
BM
25um
Safranin
HC
BM
TB
BM
25um
DAPI
BM
TB
BM
Sirius red
TB
BM
200um
Sirius red
HC
TB
BM
TB
25um
Sirius red Polarization
HC
TB
BM
TB
25um
SHG
HC
TB
BM
TB
25um
HC
HC
HC
HC
TB
TB
BM
TB
TB
BM
TB

Figure supplementary 1. First-row: High magnification images of Figure 1D. Hypertrophic cartilage area (HC) and Trabecular bone area (TB) are a continuum histological structure. At growth plate area, ATPV61B1 (VPP3) staining shows non-resorbing vessel-associated osteoclasts (VAOs) in the vicinity of blood vessels, here shown by endomucin staining. Non-resorbed cartilage cells (Dotted line squares) remains embedded within extracellular matrix at trabecular bone (TB), forming cell-matrix bars surrounded by bone marrow (BM). These embeeded cells show collagen in their periphery as shown in red at Sirius red staining and Collagen 1 immunostaining. This collagen forms oriented red-orange collagen fibers as observed under polarized light. The rest of extracellular matrix is positive for safranin staining as at growth plate.

Second-row: Details of the collagen in the periphery of representative non-resorbed cartilage cells embedded at the starting point of trabeculae. Low magnification image shows Sirius red collagen staining, note absence of staining at cartilage growth plate, and the different red staining pattern at trabecular bone and at cortical bone at the secondary ossification center. The black square corresponds to the magnified area in the rest of the images. Dotted lined squares indicate the location of non-resorbed cartilage cells embedded within trabecular bone extracellular matrix and surrounded by collagen fibers, shown as red staining in Sirius red staining, fibers at Sirius red staining under polarized light, and green fluorescent structure when visualized at two photon microscopy second harmonic generation (SHG) microscopy.

Third-row: Surface Electron microscopy (SEM) images at the same area. Images are provided from low magnification to high magnification of those areas indicated by black squares. Two areas are provided, the top one corresponds to hypertrophic chondrocytes (HC) and their cell periphery is a plane tissue with no fibers (blue arrows). Images below correspond to the following cell in the chondrocyte vertical column (White dotted lined square) which is a non-resorbed cartilage cell embedded within trabecular bone (TB) extracellular matrix and surrounded by fibrillar structures (white arrows). In the same image, the trabecular bone surface facing bone marrow (BM) shows the same fibrillar structures.

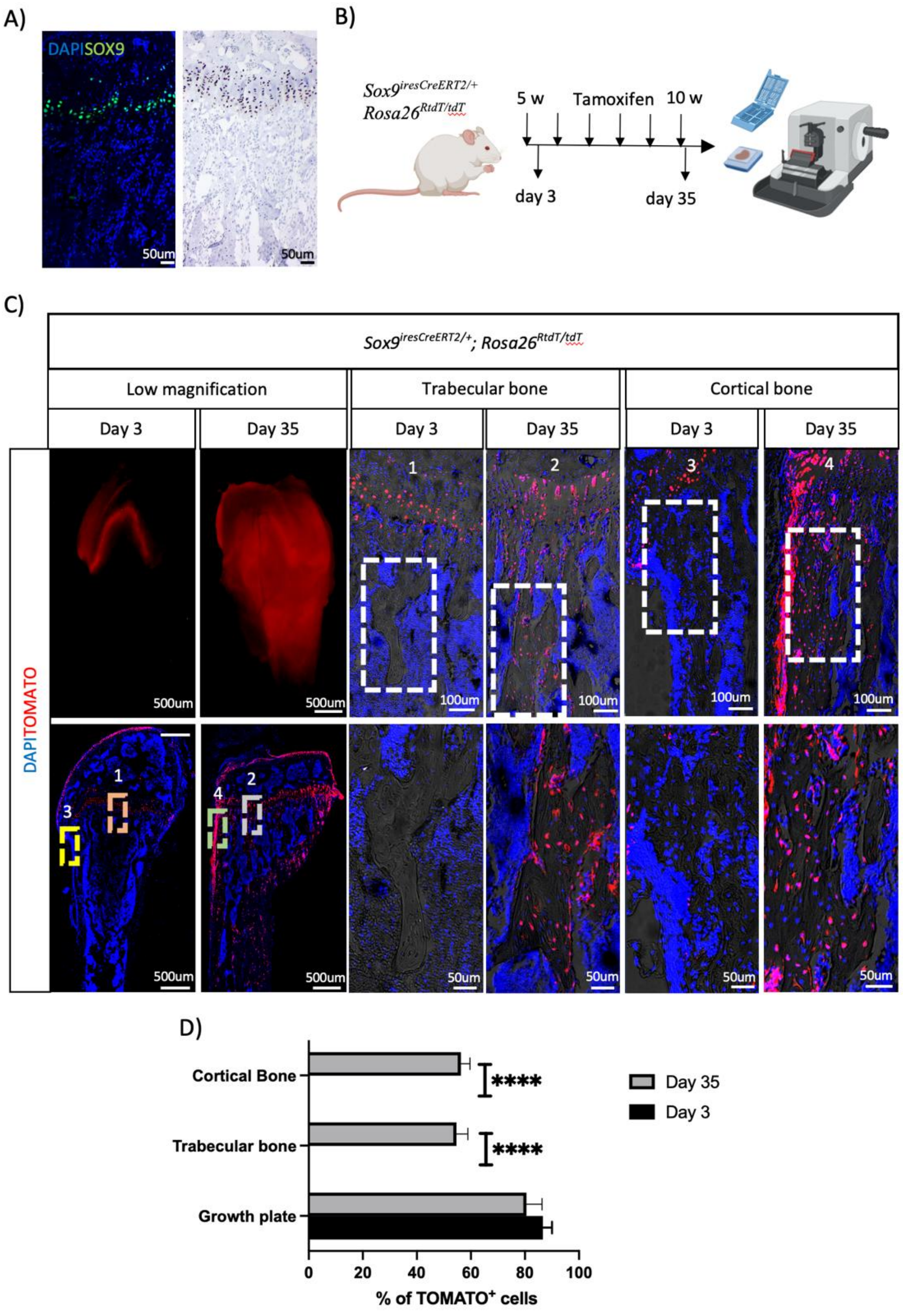

Supplementary Figure 2. Lineage tracing of SOX9⁺ cells form growth plate to trabecular and cortical bone. A) SOX9⁺ cells located at cartilage by immunostaining in 5-week-old mice tissue samples. B) Schematic of tamoxifen treatment for the induction of tomato expression. C) Representative images of Tomato fluorescence in fresh tibia tissue (stereo fluorescence microscope images), and histology immunostaining. Specific areas are numbered and shown in more detail. D) Quantification of tomato positive cells at different tissues (statistics refer to day 3 vs day 35 within the same tissue) (n=18).

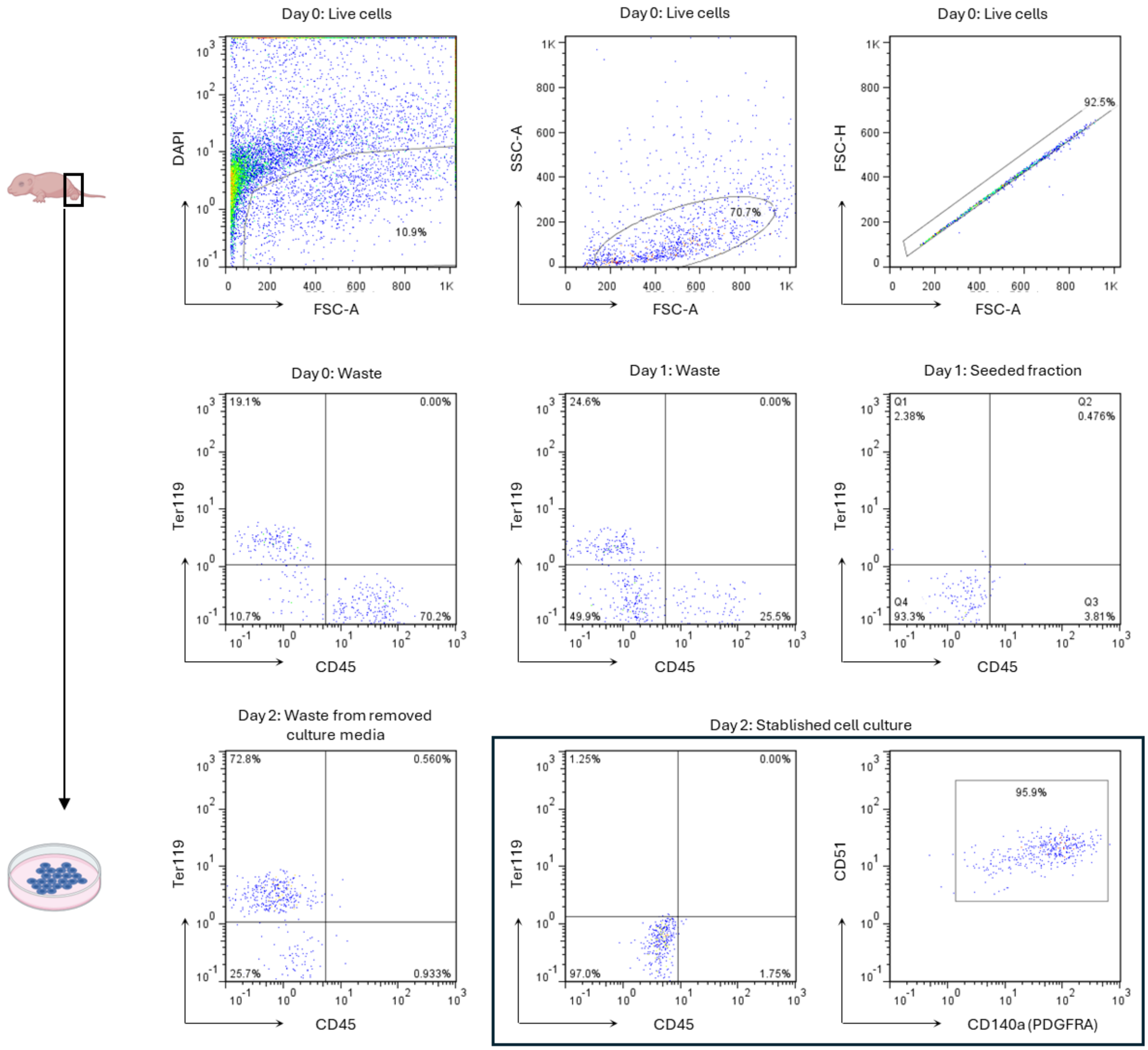

Supplementary Figure 3. Primary cell culture established from pups cartilage tissue. Phenotype characterization by Citometry. Gating strategy is provided. Then different steps of the protocol samples were recovered and analyzed.

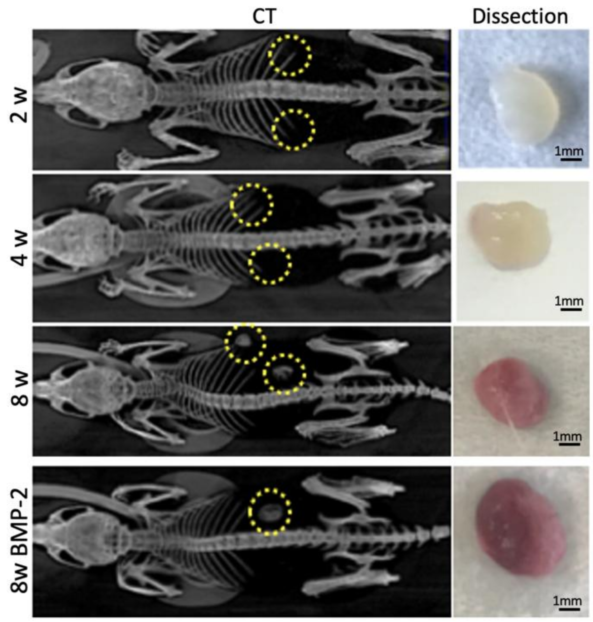

Supplementary figure 4. Computed tomography and gross morphology of scaffolds implanted without BMP-2 for 2-4-8 weeks and with rhBMP-2 for 8 weeks (n=3).

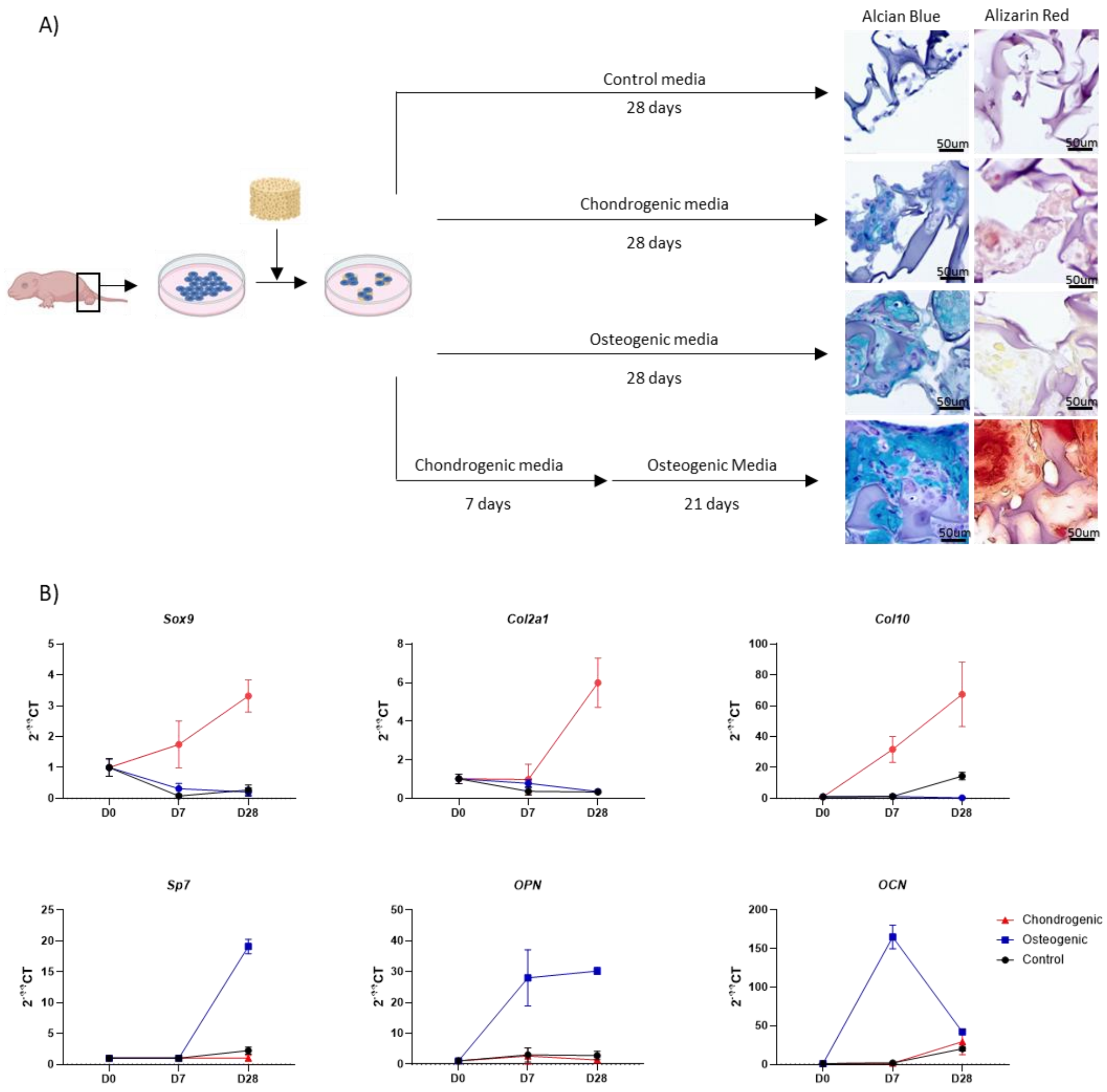

Supplementary figure 5. *In vitro* primary cartilage precursor differentiation. A) Histology data from assays done using cell-seeded scaffolds. Similar to self-assembled cell-pellet approach shown in figure 3A, a chondrogenic media treatment followed by osteogenic media serial differentiation protocol is the only one inducing matrix calcification. **B) Gene expression kinetic study of selected cartilage and bone tissue marker genes in control differentiation conditions.** As control to the data shown in figure 3B, data were also obtained from self-assembled cell-pellets treated individually with each of the differentiation media (Control, Osteogenic or Chondrogenic media) (n=3).

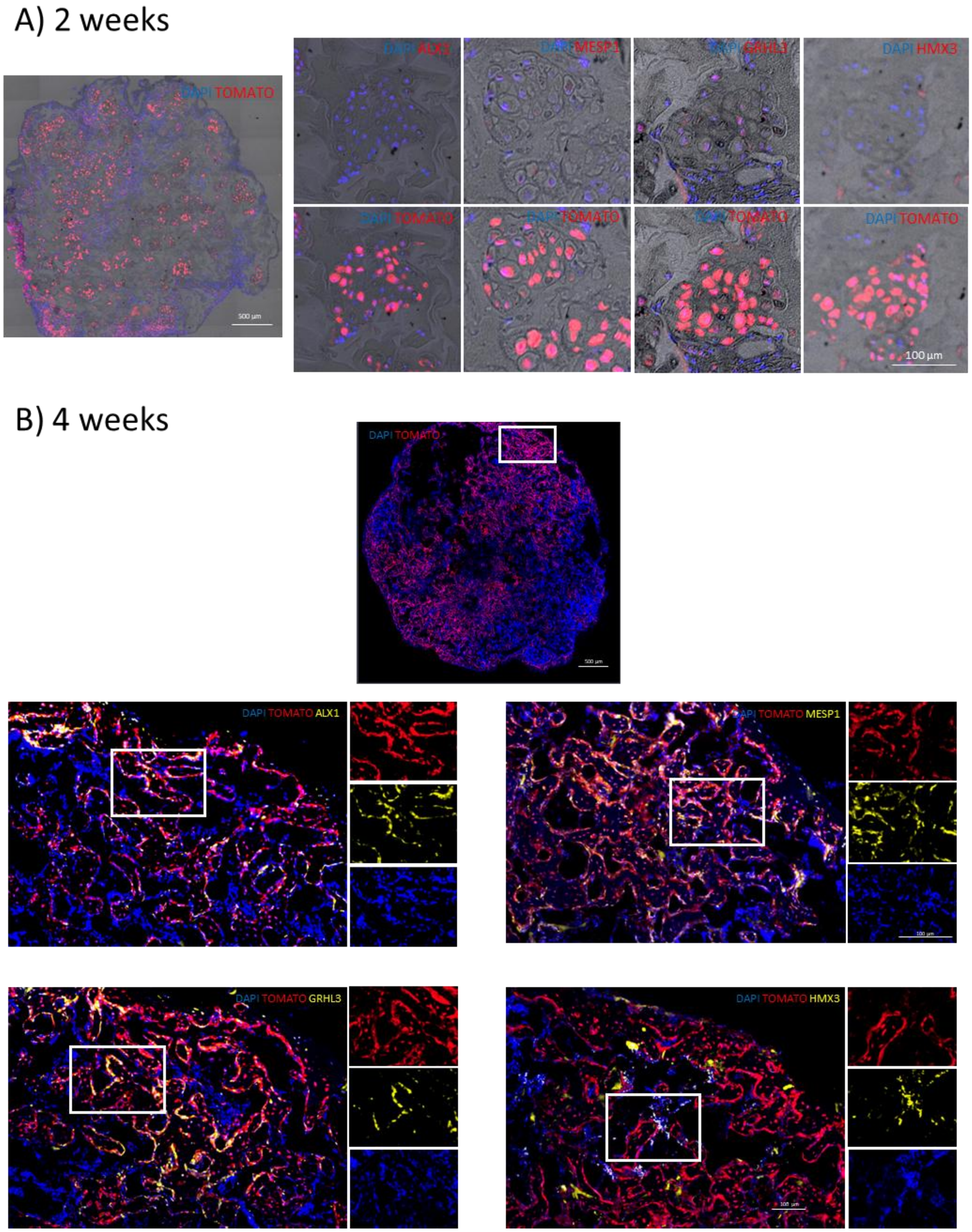

Supplementary figure 6. Doble immunostaining at bone-forming implantation model. Implants were carrier of TOMATO expressing cells. A) Samples harvested after 2 weeks of implantation **B)** Samples were harvested after 4 week of implantation (n=3 per condition).

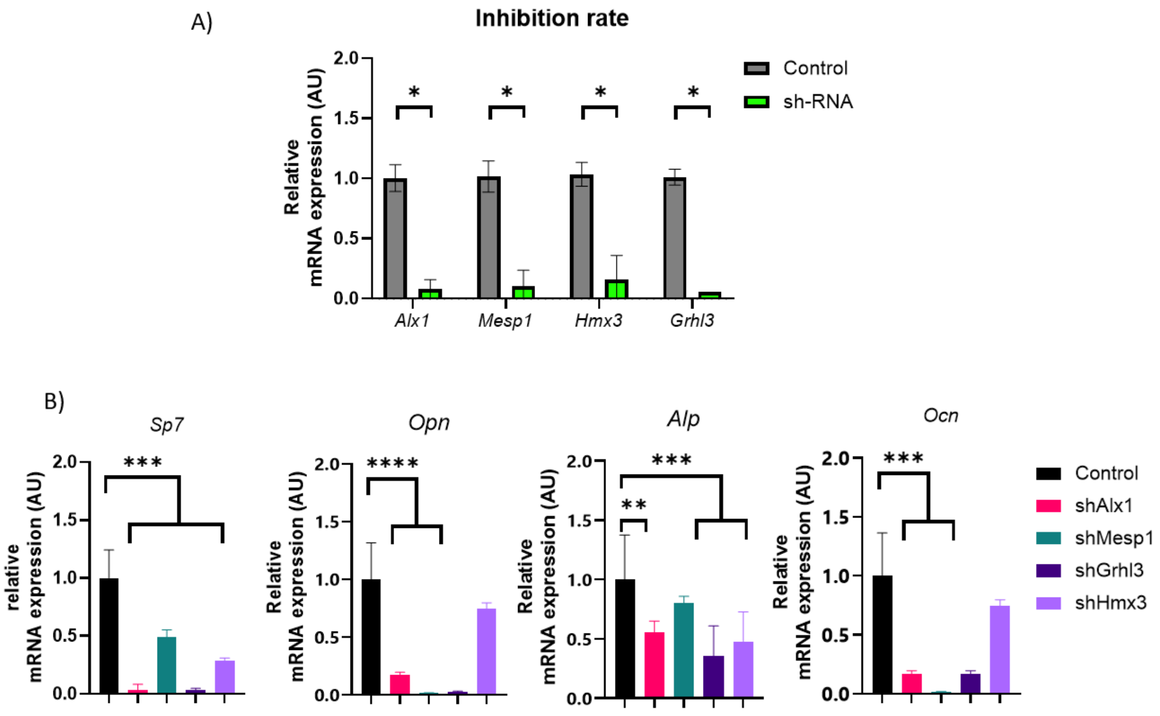

Supplementary figure 7. shRNA assays. A) Inhibition rates obtained due to shRNA lentiviral vector transfection. RNA was extracted at day 10 after transfection and antibiotic selection. B) RT-qPCR analysis of osteogenic genes expression in self-assembly cell pellets done with transfected cells and processed for *in vitro* serial differentiation protocol for 28 days (n=2 to 5).

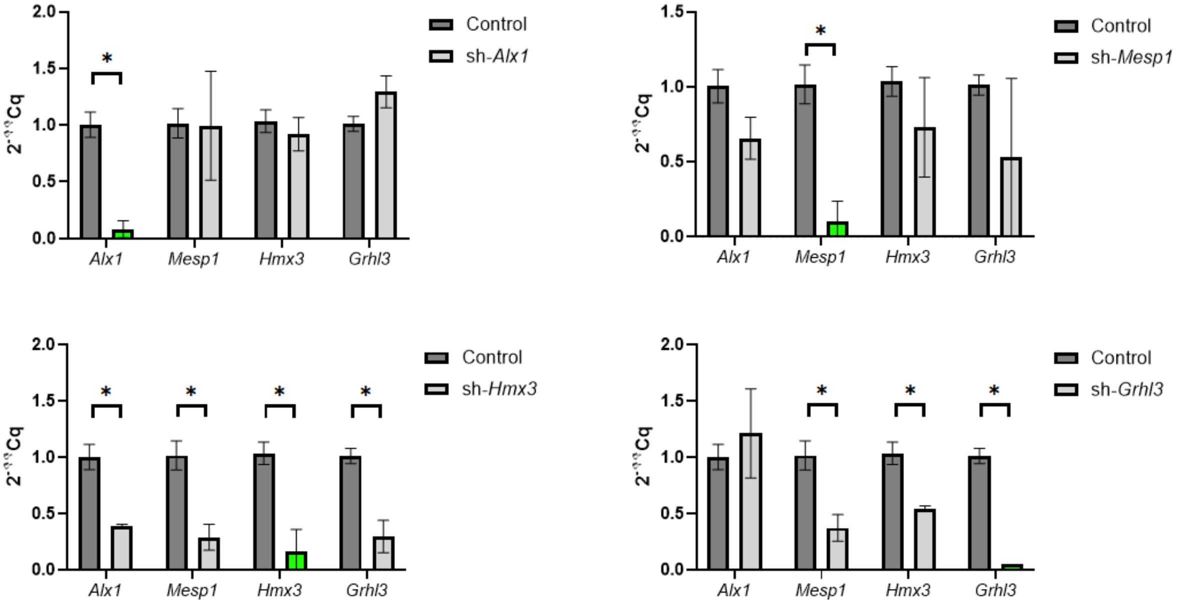

Supplementary figure 8. shRNA assays. A) Effect of specific gene silencing in the expression of the other targeted genes. When *Alx1* and *Mesp1* were silenced there was non-significative effect in the expression of other genes. When *Hmx3* and *Grhl3* were silenced, the rest of the target genes were also downregulated (n=2 to 5).

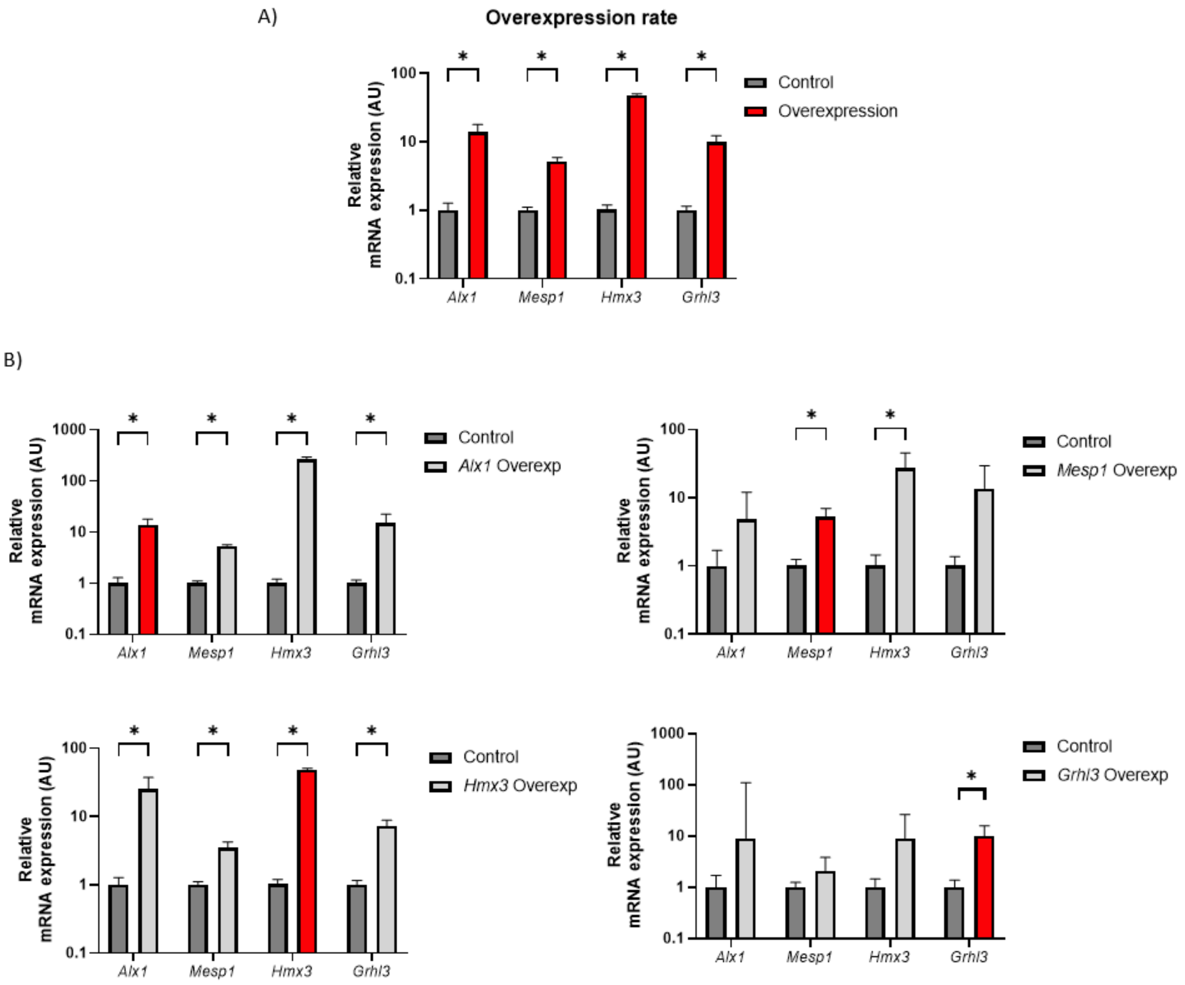

Supplementary figure 9. Overexpression assays. After Transduction and antibiotic selection, self-assembly cell pellets were done with transfected cells and processed for *in vitro* serial differentiation protocol. mRNA was extracted from samples after 7 days in chondrogenic media and 3 days in osteogenic media. A) Expression of targeted genes in each transfection approach. B) Expression of the other targeted genes. Note that, in all cases, the overexpression of the specific targeted gene followed by serial differentiation induces the same trend of overexpression in the other targeted genes, suggesting the potentiation of the differentiation process in all cases (n=2 to 5).

Extended Materials and Methods

Supplementary Table 1. Key resources:

General Reagent	Source	Identifier
1 mL Syringe with needle of 25G	Terumo, JPN	SS+01H25161
Acetic Acid, Reagent plus (R), >=99%	Merck, Ge	A6283-1L
Alcian-Blue Staining	Merck, Ge	TMS-010-C
Alizarin-Red Staining Solution	Merck, Ge	TMS-008-C
Antigen retrieval buffer	Vector Laboratories, Inc., US	H-3300
BLOXALL Endogenous Peroxidase and Alkaline Phosphatase Blocking Solution	Vector Laboratories, Inc., US	SP- 6000
Bovine Serum Albumin	Merck, Ge	A7906-10G
Carprofen	Zoetis, US	Rimadyl
Cell strainer (70 μm)	Corning, US	45352350
Collagenase D	Merck, Ge	11088882001
DAPI (4′,6-Diamidino-2-phenylindole dihydrochloride)	ThermoFisher Scientific Inc., US	D8417
Dimethyl sulfoxide	Merck, Ge	D2650-5X10ML
D-MEM+4500mg/L GLUCOSE+GLUTAMAX TMI-PYRUVATE	ThermoFisher Scientific Inc., US	A361965026
DNase 25mg	STEMCELL Technologies, Canada	7469
Donkey serum	Merck, Ge	D9663-10ML
Ethanol	Scharlab, ES	ET0003005P
Eukitt® Quick-hardening mounting medium	Merck, Ge	03989-100ML
FBS HEAT INACT. S.AMERICAN(CE) 500ML	ThermoFisher Scientific Inc., US	A310500064
Fibrinogen, human type I from human plasma	Merck, Ge	F3879-100MG
FisherbrandTM Borosilicate Glass Rectangular Coverslips	ThermoFisher Scientific Inc., US	12373128
Fluorescent mounting media	Dako, Agilent Technologies, US	S3023
Formalin 10%	Merck, Ge	HT501128-4L
Gelfoam Pack 12cmx12cm 4 Pieces	Pzifer, US	43521
Goat serum	Merck, Ge	G9023-5ML
GoTaq(R) 2-Step RT-qPCR	Promega, US	A6010
Hematoxylin and Eosin Staining kit	Abcam, UK	ab245880

High Sensitivity D1000 ScreenTape Assay	Agilent Technologies, US	HS-D1000
Illumina Stranded mRNA Prep, Ligation	Illumina, US	20040534
ImmPACT DAB	Vector Laboratories, Inc., US	SK-4105
Invitrogen™ Qubit™ dsDNA Quantification Assay Kit	ThermoFisher Scientific Inc., US	Q32851
Isoflurane	Abbott, US	B506
iTaq Universal SYBR Green Supermix	Merck, Ge	D61725121
L-glutamine (200 mM)	ThermoFisher Scientific Inc., US	A325030024
MEM α, nucleosides	ThermoFisher Scientific Inc., US	A322571020
MesenCult Osteo Stim. Kit (Mouse)	STEMCELL Technologies, Canada	5404
MesenCult-ACF Chondro Differentiation Kit	STEMCELL Technologies, Canada	5455
Monarch™ Total RNA Miniprep Kit	New England Biolabs, US	174T2010S
Osteosoft Mild decalcifier-solution	Merck, Ge	1017281000
Paraffin	Leica, DE	39602004
PBS PH 7,4 (1X)	Corning, US	5521-030-CV
PBS, 1X Powder Concentrate 98.9GR	ThermoFisher Scientific Inc., US	10316743
Penicillin-streptomycin	ThermoFisher Scientific Inc., US	11528876
Polybrene Infection / Transfection Reagent	Merck, Ge	TR-1003-G
Puromycin dihydrochloride	SantaCruz Biotechnology, US	sc-108071
Recombinant human bone morphogenetic protein-2 (rhBMP-2)	Noricum, Spain	rhBMP-2
Safranin-O Staining	Merck, Ge	TMS-009-C
Sirius red/Fast Green Collagen Staining kit	Amsbio, US	9046
Sterile tissues	-	-
Sudan Black	Merck, Ge	S2380
Superfrost microscope slides	ThermoFisher Scientific Inc., US	15438060
Surgical material	multiple	multiple
Tamoxifen	Merck, Ge	T5648
Thrombin from human plasma	Merck, Ge	T8885-10VL
Triton X-100	Merck, Ge	T9284-500ML

Trypan Blue solution	ThermoFisher Scientific Inc., US	T8154-20ML
Trypsin-EDTA (0.25%), phenol red	ThermoFisher Scientific Inc., US	11528876
U-Bottom 96 well plate	Corning, US	353077
Ultra-Low Attachment Multiple Well Plates	Corning, US	3473 or 3471
Vectasain Elite ABC kit Peroxidase	Vector Laboratories, Inc., US	PK-6100
Xylene	Bio-Optica, IT	06- 1304Q
**Antibodies**	**Source**	**Identifier**
Donkey anti-Goat IgG (H+L) Cross-Adsorbed Secondary Antibody, Alexa Fluor 647	ThermoFisher Scientific Inc., US	A-21447
Donkey anti-Mouse IgG (H+L) Highly Cross-Adsorbed Secondary Antibody, Alexa Fluor™ 594	ThermoFisher Scientific Inc., US	A-21203
Donkey anti-Rabbit IgG (H+L) Highly Cross-Adsorbed Secondary Antibody, Alexa Fluor™ Plus 488	ThermoFisher Scientific Inc., US	a-32790
Goat anti-GFP, polyclonal antibody	Abcam, UK	ab6673
Goat anti-Mouse IgG (H+L) Cross-Adsorbed Secondary Antibody, Alexa Fluor 594	ThermoFisher Scientific Inc., US	A-11005
Goat anti-Rabbit IgG (H+L) Cross-Adsorbed Secondary Antibody, Alexa Fluor 488	ThermoFisher Scientific Inc., US	A-11008
Goat anti-Rabbit IgG (H+L) Cross-Adsorbed Secondary Antibody, Alexa Fluor™ 647	ThermoFisher Scientific Inc., US	A-21244
Goat anti-tdTomato, polyclonal antibody	Biorbyt, UK	orb182397
Horse Anti-Mouse/Rabbit/Goat IgG Antibody (H+L) (Universal Pan-Specific), Biotinylated	Vector Laboratories, Inc., US	BA-1300-2.2
Mouse and Perilipin, Monoclonal antibody	Progen	690156s
Mouse anti-Alx1 [96K], monoclonal antibody	SantaCruz Biotechnology, US	sc-130416
Mouse anti-Grhl3 [C-12], monoclonal antibody	SantaCruz Biotechnology, US	sc-398838
Mouse anti-Mesp1 [JH.12], monoclonal antibody	SantaCruz Biotechnology, US	sc-130461
Mouse anti-VEGF Monoclonal Antibody (JH121)	ThermoFisher Scientific Inc., US	MA5-13182
Rabbit anti Osteocalcin, polyclonal antibody	Acris	Bs-0470R
Rabbit Anti-ATP6V1B2 (VPP3), polyclonal antibody	Abcam, UK	ab73404
Rabbit anti-Hmx3, polyclonal antibody	Biorbyt, UK	orb693637
Rabbit anti-Sox9, polyclonal antibody	Bioss Antibodies Inc., US	bs-10725R
Rabbit Col1, monoclonal antibody	Abcam	ab260043
Rat anti Ter-119 Antibody, anti-mouse, APC	Miltenyi Biotec, Ge	130-102-290

Rat anti-mouse CD202b (TIE2) Monoclonal Antibody (TEK4), APC	ThermoFisher Scientific Inc., US	17-5987-82
Rat anti-mouse CD51 (Integrin alpha V) Monoclonal Antibody (RMV-7), PE	ThermoFisher Scientific Inc., US	12-0512-81
Rat anti-Endomucin [V.7C7], monoclonal antibody	SantaCruz Biotechnology, US	sc-65495
recombinant human anti-mouse CD140a (PDGFRA) Antibody, APC-Vio® 770, REAfinity	Miltenyi Biotec, Ge	130-125-991
recombinant human anti-mouse CD45 Antibody, anti-mouse, FITC, REAfinity™	Miltenyi Biotec, Ge	130-110-796
**Lentiviral particles**	**Source**	**Identifier**
Alx1 (m) Lentiviral Particles Lenti-ONE™	GEG tech, France	lenti-ODS230622004
Alx1 shRNA (m) Lentiviral Particles	SantaCruz Biotechnology, US	sc-141035-V
copGFP Control Lentiviral Particles	SantaCruz Biotechnology, US	sc-108084
Grhl3 (m) Lentiviral Particles Lenti-ONE™	GEG tech, France	lenti-ODS230622002
Grhl3 shRNA (m) Lentiviral Particles	SantaCruz Biotechnology, US	sc-145762-V
Hmx3 (m) Lentiviral Particles Lenti-ONE™	GEG tech, France	lenti-ODS230622003
Hmx3 shRNA (m) Lentiviral Particles	SantaCruz Biotechnology, US	sc-146056-V
Mesp1 (m) Lentiviral Particles Lenti-ONE™	GEG tech, France	lenti-ODS230622001
Mesp1 shRNA (m) Lentiviral Particles	SantaCruz Biotechnology, US	sc-149373-V

Animals

All animal experiments were approved by the ethical committee and local competent authorities, under the project licenses no. 70/8560, PP8826065 and PRO-AE-SS-171 (competent authorities: Departamento de promoción económica, medio rural y equilibrio territorial, Diputación de Gipuzkoa, and; UK Home Office animals (scientific procedures) act 1986).

Assays were performed in compliance with relevant guidelines (Directive 2010/63/EU) and following the ARRIVE guidelines and recommendations. All mouse lines used in this studies have been previously described: C57BL/6J inbred strain*, MGI:* 3028467*; Sox9*$^{tm1(cre/ERT2)Haak}$ targeted tamoxifen inducible insertion, MGI: 4867441 (50); *R26R*^{tomato} targeted mutation, MGI: 3809523 (51, 52); *Col2a1*-Cre^{ERT} targeted tamoxifen inducible insertion, MGI: 3665440; R26R^{EYFP} MGI:2449038; NOD SCID, Immunodeficient spontaneous mutant strain, MGI:2163032. The transgenic mice used in this study were generated by crossing and breeding these strains.

All animals were housed in ventilated cages and fed with standard diet *ad libitum*. Genotyping was performed using Transnetyx. When needed, tamoxifen was used to induce the expression of Cre activity. Tamoxifen solution (20mg/ml) administration was done by oral gavage at 5 mg/25 g body weight. For neonatal induction in pups, tamoxifen was administered subcutaneously at a dosage of 0.25 mg per gram of body weight (0.25 mg/g body weight) once daily for two consecutive days. Alternatively, 4-OHT (1uM during 24h) was added to culture media of cells in culture.

For experimental research, male and female animals were allocated randomly to each experimental group with a minimum of 3 animals per group. The groups and n are mentioned in the text or figure legends or is shown in each figure panel or graphic as individual dots.

Bone tissue characterization

Tibia, femur and implant samples were imaged by computed tomography. µCT scans were performed using a SkyScan-1176 µCT scanner (Bruker MicroCT, Kontich, Belgium).The X-ray source was operated at 40kV and 600µA, no filter was used. The scans were made over a trajectory of 180º with a 0.4º step size with a 9.01µm pixel size. The images were reconstructed using nRecon (Bruker MicroCT). CT scans were performed using the MOLECUBES (Ghent, Belgium) X-CUBE (CT) systems. A spiral high-resolution scan was performed (140mA intensity, 40 kV voltage). CT data was reconstructed through an iterative image space reconstruction (ISRA) algorithm, with 200 µm voxel size. PMOD (Bruker) image quantification software was used for the analysis of CT images. Volume of interest (VOI) was selected to delimit the implant. A threshold in the grayscale was used to define the calcified tissue within the VOI and calculate its volume.

Fixation with Formalin was followed by decalcification with Osteosoft Mild decalcifier solution, paraffin embedding and sectioning at 4 µm using a microtome (HistoCore BIOCUT Manual Rotatory Microtome, Leica Biosystems). After deparaffination, sections were used for multiple procedures as follows.

Surface electron microscopy (SEM) Deparaffined sections were dried at RT and stored at desiccator. Samples were imaged in a Field Emission Scanning Electron Microscope JSM-IT800 (JEOL, Tokyo, Japan) operated at 1.00kV acceleration voltage and probe current of 3.0. Secondary electron detector imaging at low acerating voltage and low current allows topography images with high surface detail of non-conductive samples reducing beam-induced damage on the sample and edge effect artifacts.

Second Harmonic generation (SHG). To detect structured collagen, deparaffined sections were mounted and the Second Harmonic Generation nonlinear optical process was observed using a Zeiss LSM 880 NLO multiphoton laser microscope coupled with a Mai Tai Deep See multiphoton laser. Imaging was performed with a C Apochromat 40× water objective (1.20 numerical aperture). Big2 detectors (Non-Descanned Detectors) were used for image acquisition in ranges 380-430nm

(Collagen SHG) and 575–610 nm (Background), with excitation at 820 nm at 1% power. The image pixel size was set to 0.10 micrometers, with 8-bit resolution.

Staining. Deparaffined sections were used for Hematoxylin and Eosin H&E staining (Abcam, Cat.# ab245880); Alcian blue staining with Hematoxylin counterstaining (Merck, Cat.# TMS-010-C and Abcam, Cat.# ab245880); Safranin-O (Merck, TMS-009-C), Sirius red/Fast Green Collagen (Amsbio, 904), Alizarin red (Merck, TMS-008-C) mounted and examined using an optical microscope equipped with a simple add-on polarizer and a digital imaging system (BA210 Digital Microscope, Motic).

Immunostaining. Mouse non-cross-reactive antibodies used for immunofluorescence and/or immunohistochemistry staining (See supplementary table 1)

Immunofluorescence (IFF): Deparaffined sections were used for antigen retrieval and permeabilization by incubating the slides in a steamer for 40 minutes in retrieval buffer. Upon reaching room temperature (RT), sections were washed five times for 10 minutes each in PBS containing 0.1% Triton X-100.To block nonspecific binding, sections were incubated for 30 minutes at RT in blocking buffer composed of 1% BSA, 5% donkey or goat serum, and 0.1% Triton X-100 in PBS. Slides were covered with parafilm to prevent evaporation during incubation. Following blocking, excess buffer was gently removed, and tissue boundaries were delineated with a hydrophobic PAP pen. Forty microliters of diluted primary antibody (1:200 to 1:1000 depecing on the antibody) were applied to the target sections, while control sections received 40 µl of a diluent solution (PBS, 0.1% Triton X-100, 0.2% BSA, 1% serum). Slides were sealed with parafilm and incubated overnight at 4 °C in a humidified chamber. The following day, slides were washed three times for 5 minutes in PBS-Triton 0.1%. Subsequently, 40 µl of diluted secondary antibody (1:500) was applied to each section, and slides were incubated for 1 hour at RT in the dark, again within a humidified chamber. After incubation, slides were washed three times in PBS. To reduce erythrocyte autofluorescence, Sudan Black staining was performed for 10 minutes and then slides were washed three times in PBS. Sections were then mounted with DAPI-containing mounting medium for nuclear counterstaining.

Stained samples were imaged using a ZEISS Axio Observer fluorescence microscope. Specific protein localization was visualized based on the fluorescent signal from the antibody-conjugated fluorophores.

Immunohistochemistry (IHC): Antigen retrieval was achieved by heat treatment for 40 minutes. Endogenous peroxidase activity was blocked with BLOXALL™ and tissues were washed with PBS containing 0.1% Triton X-100. Non-specific binding was prevented by incubating the sections with a blocking buffer (1% BSA, 5% serum, 0.1% Triton X-100) for 30 minutes. Primary antibodies were applied to the sections, and control samples were incubated with diluent. After overnight incubation at 4°C, sections were incubated with the biotinylated secondary antibody for 1 hour at room temperature. Signal amplification was performed using the VECTASTAIN® Elite ABC kit. After signal development with ImmPACT® DAB, counterstaining was done with hematoxylin, followed by dehydration in ethanol and xylene. Finally, slides were mounted with Eukitt® Quick-hardening medium and examined using an optical microscope with a digital imaging system (BA210 Wifi Digital Microscope, Motic).

Primary cartilage progenitor cell harvesting

Murine cartilage progenitors were obtained from of different mouse strains. Cells were isolated from 5 or 6 days-old pups following the procedure described by Gosset *et al*. (53). Briefly, Heads and condyles from femurs and heads from tibiae were separated using a scalpel and incubated with digestion solution (MEM α, nucleoside (Gibco, Cat.# 22571020) containing 1% penicillin streptomycin (Gibco, Cat.# 15070063) and Collagenase D 3 mg/mL (Merck, Cat.# 11088882001) for 45 min at 37ºC. With a pipette, the tissue was dissociated from the cartilage pipetting up and down until cartilage pieces were cleared. These cartilage pieces were transferred to a petri dish and incubated overnight with diluted digestion solution (0,5 mg/mL of Collagenase D). On day 2, media and cell were retrieved and after adding an equal volume of cold complete medium, the pieces were dissociated pipetting up and down until a suspension of isolated cells was obtained. Cells were filtered through a cell strainer (70 µm) (Corning, Cat.# 00431751) to remove undigested tissue or debris and centrifuged for 10 mins, at 1250 rpm at 4ºC. Supernatant was removed and cells

were resuspended in complete culture medium. Cells were centrifuged and resuspended again and plated on culture dishes overnight. A fraction of these cells was separated and characterized in a MACSQuant® Analyzer 10 Flow Cytometer using the following anti-mouse antibodies (Dilution 1:1000 in all cases): CD45 (Miltenyi, Cat.# 130-110-796); Ter-119 (Miltenyi, Cat.# 130-102-290); CD202b (TIE2) (Thermo Fisher Scientific, Cat.# 17-5987-82); CD140a (PDGFRA) (Miltenyi, Cat.# 130-125-991); CD51 (Integrin alpha V) (Thermo Fisher Scientific, Cat.# 12-0512-81). The rest was frozen down for further studies. Cell suspensions were centrifuged at 200g for 5 minutes, resuspended in PBS with 2% FBS, and incubated with fluorescent antibodies for 15–30 minutes. After adding additional PBS with 2% FBS, samples were centrifuged again, and resuspended in PBS with 2% FBS and DAPI. The samples were filtered with a 100 µm mesh cap and loaded into a flow cytometer for analysis based on fluorescent properties. FlowJO software was used for further population analysis.

Scaffold preparation

Cell carrier implants were prepared as previously described (65, 66). Briefly, 20x60x7 mm Gelfoam gelatin sponges (Pfizer, Cat. # 0009-0323-01) were sectioned into 128 pieces (4x4x4 mm each), washed with 70% ethanol (Scharlab, Cat.# ET0003005P) and rehydrated in sterile phosphate buffered saline (PBS) (Gibco, Cat.# 14040). Cells were diluted in culture media at $1x10^6$ cells/ml and 100µl ($1x10^5$ cells) were carefully inoculated in each scaffold using a 1mL syringe (Braun, Cat.# 9161406V) with 25G needle (Braun, Cat.# 9186166). Cell-seeded scaffolds were transferred to polystyrene ultra-low attachment 24-well plates (Corning, Cat. # 3473) and kept in cell culture conditions for 3-5 hours. Then, culture media was added, and scaffolds were maintained in cell culture conditions for further studies.

In vivo subcutaneous implantation

Cell-seeded scaffolds cultured for 3-7 days were transferred to clean well and supplemented with 5 µL of 5 µg/µL rhBMP-2 (Noricum, Cat. # rhBMP-2) reconstituted in 50 mM acetic acid (Fluka, Cat. # 27225). Then, 30 µL of 2% $CaCl_2$ reconstituted thrombin from human plasma (Merck, Cat. # T8885) and 30 µL of water reconstituted fibrinogen from human plasma (Merck, Cat. # F3879) were incorporated. Clotting was allowed during 10 minutes in cell culture conditions before proceeding with *in vivo* implantation.

Ten-week-old healthy NOD-SCID (Charles River laboratories, strain code 394) mice were used for *in vivo* implantation studies. The animals were randomly allocated to the following groups:

Sample size was stablished at n=3 per group and two implantation times and according to the primary outcome measure (Histology). The total number of used animals was 24. No exclusions were performed. Group allocation was blinded at the outcome assessment and data analysis steps. Anesthesia was induced with 2.5% isoflurane and O_2 at 2-4%. PBS reconstituted analgesic and anti-inflammatory Carprofen (Zoetis, Rimadyl) (5 µg/g of animal) was administered subcutaneously. A wide section of fur from the back was shaved. Then, skin was sterilized twice with Povidone-iodine (Mylan, Cat. # Betadine). For each scaffold implantation, 0.5 cm incision was made in the skin located just on top of the spine of the animal. With forceps, and using the skin-generated incision, a pocket under the skin was made down the side of the animal. A scaffold was inserted, making sure it was placed deep within the pocket, and then the incision was stapled and cleaned with Povidone-iodine. Anesthesia was removed and animals were supervised until total recovery. PBS reconstituted analgesic and anti-inflammatory Carprofen (5 µg/g of animal) was administered subcutaneously every 24 h during 3 days after surgery. Carbon dioxide (CO_2) overdose was used to euthanize the animals.

In vitro cell culture studies

Self-assembled cell pellets were formed by centrifugation of 2,5x10^5 to 1x10^6 cells at 200G for 5 min. Long-term cell cultures were done in cell pellets and cell-seeded scaffolds. Differentiation media were: MesenCult-ACF Chondro Differentiation Kit (Stem Cell Tech, 5455); MesenCult Osteo Stimulation Kit (Stem Cell Tech, 5404). At stablished endpoints, samples intended for histological analysis were fixed and processed for paraffin-embedding and staining as described above, while samples intended for gene expression studies were processed as follows.

Gene expression studies

Cell pellet samples were disrupted and homogenized. RNeasy Plus Mini Kit (Qiagen, Cat. # 74134) was used to remove genomic DNA (gDNA) and isolate total Ribonucleic acid (RNA) according to the producer's instructions. RNA concentration was measured using a Nanodrop spectrophotometer (Thermo fisher Scientific).

For **Quantitative Real-Time PCR (**qPCR) assay, primers for target genes were designed using primer-BLAST primer design tool (NIH, USA) (Supplementary table 2) and purchased (Merck). RNA was reverse transcribed to cDNA and prepared for RT-PCR reaction using the GoTaq(R) 2-Step RT-qPCR kit (Promega Biotech Iberica, Cat. # A6010) and based on the manufacturer's indications. Real time PCR reaction was performed using a CFX Connect Real-time PCR detection system (Bio-rad) and differential gene expression was calculated by using the $2^{-\Delta\Delta Ct}$ method. Gene expression levels of target genes were measured by normalization to the housekeeping gene *GAPDH* exploiting control samples as calibrators. Data are provided normalized to data of cells seeded in control cell culture standard plastic surface. All the reactions were conducted in triplicates.

For **RNA-sequencing**, roughly 150 ng of high-quality total RNA (RIN >8) was used for the transcriptomic assays using Illumina's Stranded mRNA Prep ligation according to the manufacturer's instructions. Briefly, oligo(dT) magnetic beads were used to capture and purify poly-adenylated mRNA molecules from total RNA. The

purified mRNA was then fragmented and reverse-transcribed to cDNA using random primers. A second strand cDNA synthesis step removed the RNA template while incorporating dUTP in place of dTTP in order to preserve strand specificity. Next, double-stranded cDNA was A-tailed, then ligated to Illumina anchors bearing T-overhangs. PCR-amplification of the library allowed the barcoding of the samples with 10bp dual indexes and the completion of Illumina sequences for cluster generation. Libraries were quantified with Qubit dsDNA HS Assay Kit and their profile was examined using Agilent's HS D1000 ScreenTape Assay. Sequencing was carried out in an Illumina NextSeq2000 using paired-end, dual-index sequencing (Rd1: 59 cycles; i7: 10 cycles; i5: 10 cycles; Rd2: 59 cycles) at a depth of 30 million reads per sample.

Further RNA-sequencing data analysis were conducted as previously described (56). We used HISAT2 (57) to align the RNA-seq reads to the human reference genome hg38, and Cufflinks (58) to annotate them. We calculated the counts of aligned reads to each gene with HTSeq (59). We merged the transcriptomics results into a single text file and used it in the downstream analysis in Matlab. We equalized the data and stabilized them through the log2 transform of the data plus one; calculated the average values for each group of replicates; selected the Differentially expressed genes (DEGs) whose absolute value of difference of mean values between the two groups was less than a selection threshold θ_{DEG}=1 of fold change in log2 scale; selected the statistically significant DEGs using the Student's t-test with a significance threshold α_{DEG}= 0.05. DEGs sets were used to perform Gene Ontology (GO) enrichment analysis (http://geneontology.org). Additionally, Gene Set Enrichment Analysis was performed with GSEA (http://software.broadinstitute.org/gsea/msigdb). The scatter plots, principal component analysis, GO and GSEA were performed using in-house functions developed in Matlab (MathWorks). Transcriptional regulatory networks were obtained using String database of known and predicted protein-protein interactions (http://string-db.org).

Supplementary Table 2. Primers:

Name	Sequence (5'-3')	Tmº	GC content (%)	Product length
ACTB-F	TGAGCTGCGTTTTACACCCT	59.8	50	231
ACTB-R	TTTGGGGGATGTTTGCTCCA	59.8	50	231
HPRT1-F	CAGTCCCAGCGTCGTGATTA	59.8	55	168
HPRT1-R	TGGCCTCCCATCTCCTTCAT	60.3	55	168
GAPDH-F	ATGACATCAAGAAGGTGGTG	60	45.00	177
GAPDH-R	CATACCAGGAAATGAGCTTG	59,7	45.00	177
SOX9-F	GAGGAAGTCGGTGAAGAACG	63,8	55.00	163
SOX9-R	CTGAGATTGCCCAGAGTGCT	64,6	55.00	163
COL10-F	ACCCCAAGGACCTAAAGGAA	63,4	50.00	146
COL10-R	CCCCAGGATACCCTGTTTTT	63,7	50.00	146
COL2A1-F	ACTGGTAAGTGGGGCAAGAC	62,9	55.00	115
COL2A1-R	CCACACCAAATTCCTGTTCA	63,4	45.00	115
OSTEOCALCIN-F (Bgalp1,Bglap2 and Bglap3)	CAAGTCCC ACACAGCAGCTT	65,5	55.00	371
OSTEOCALCIN-R (Bgalp1,Bglap2 and Bglap3)	AAAGCCGAGCTGCCAGAGTT	67,4	55.00	371
OPN-F	CACTCCAATCGTCCCTAC	58	55.56	157
OPN-R	AGACTCACCGCTCTTCAT	57,4	50.00	157
SP7-F	GGAAGGGTGGGTAGTCATTTG	64,3	52.38	271
SP7-R	TCCTCTCTGCTTGAGGAAGAAG	63,9	50.00	271
ALX1-F	GGAGACGCTGGACAATGAGT	64,3	55	673
ALX1-R	AGGCGAGTGAGAGTAAGGTG	61,1	55	673
HMX3-F	CCCATCCTCTACCACGAGAA	64	55	109
HMX3-R	AGTAGTAGACTGGGTGCGG	59,4	57,8	109
GRHL3-F	GCAAGCGAGGCATCCTGGTTAA	71	54,5	61
GRHL3-R	ACGTGGTTGCTGTAGTGTTGG	64,9	52,3	61
MESP1-F	CCGCCTGCCTACCCTAGAC	65,9	68,4	122
MESP1-R	CTGAAGAGCGGAGATGAGGGA	68	57,1	122

Cell transfections:

Cell transfection was performed with lentiviral particles for gene silencing or stable transgene expression.

The gene silencing approach involved a pool of 3 target-specific lentiviral vector plasmids each of them encoding 19-25 nt (plus hairpin) shRNAs designed to knock down gene expression of each gene of interest. Each plasmid contained puromycin resistance gene for the selection of cells stably expressing shRNA. For setting up the transfection and selection conditions cop-GFP control lentiviral vector was used and puromycin titration kill curve was performed.

Design and production of lentiviral vectors for the stable transgene expression were achieved by GEG Tech. The cDNA sequence of each target was cloned at integrative lentiviral vector pseudotyped VSVg. The cDNA sequences were linked to the neomycin resistance marker with a P2A sequence and these ORFs were under the control of the PGK promoter. See supplementary table 1 for the list of specific lentiviral particles used.

Cells were transfected by spinoculation. 200.000 cells were resuspended in a volume of 1ml of complete media containing lentiviral vectors (5 MOI) and Polybrene 8 ug/ml (Merck Cat.# TR-1003-G), followed by centrifugation at 200g at RT for 20 min, and incubation at 37_C, 95% humidity, 5% CO2 for 4–6 h. Afterwards supernatant was discarded, cells were resuspended in complete media and seeded in a 25 cm^2 cell culture flask with fresh complete media. At 24 h culture medium was removed and replaced with media supplemented with the required selection antibiotic.

Statistics

All data were plotted and statistically analyzed using the GraphPad Prism software (La Jolla, CA, USA). In all cases, data normality was verified using the Shapiro–Wilk test and a normal QQ plot was assessed before statistical analysis using parametric tests (t-test, ANOVA) and Tukey’s multiple comparisons tests.
